# Discovering and harnessing oxidative enzymes for chemoenzymatic synthesis and diversification of anticancer camptothecin analogues

**DOI:** 10.1038/s42004-021-00602-2

**Published:** 2021-12-16

**Authors:** Tuan-Anh M. Nguyen, Trinh-Don Nguyen, Yuen Yee Leung, Matthew McConnachie, Oleg Sannikov, Zhicheng Xia, Thu-Thuy T. Dang

**Affiliations:** 1 Irving K. Barber Faculty of Science, Department of Chemistry, University of British Columbia, 3427 University Way, Kelowna, BC V1V 1V7 Canada; 2 Faculty of Science, Department of Chemistry, University of British Columbia, 2036 Main Mall, Vancouver, BC V6T 1Z1 Canada

**Keywords:** Oxidoreductases, Biocatalysis

## Abstract

Semi-synthetic derivatives of camptothecin, a quinoline alkaloid found in the *Camptotheca acuminata* tree, are potent anticancer agents. Here we discovered two *C. acuminata* cytochrome P450 monooxygenases that catalyze regio-specific 10- and 11-oxidations of camptothecin, and demonstrated combinatorial chemoenzymatic C-H functionalizations of the camptothecin scaffold using the new enzymes to produce a suite of anticancer drugs, including topotecan (Hycamtin®) and irinotecan (Camptosar®). This work sheds new light into camptothecin metabolism, and represents greener approaches for accessing clinically relevant camptothecin derivatives.

## Abstract (web summary)

Camptothecin derivatives are precursors of potent anticancer agents, but their biosynthesis remains largely unknown. Here two cytochrome P450 monooxygenases are shown to regiospecifically oxidize camptothecin, yielding 10- and 11- hydroxylated derivatives, which are subsequently used to produce a suite of known anticancer drugs and derivatives.

## Introduction

**Fig. 1:**
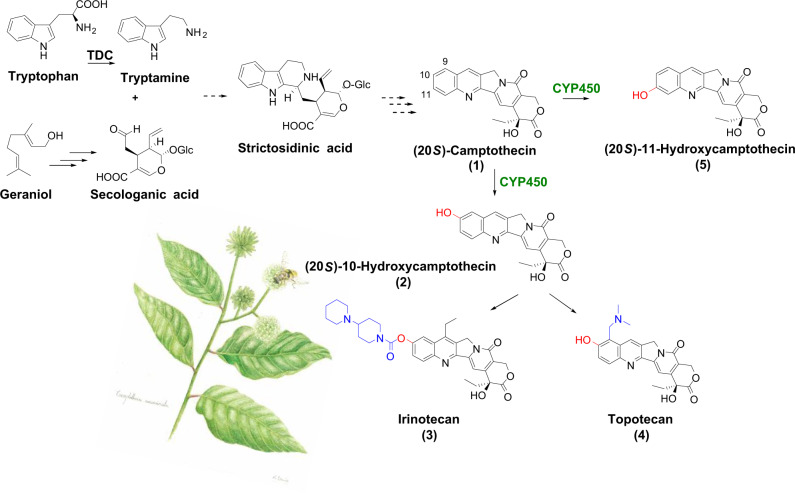
Camptothecin biosynthesis in *C. acuminata*. The oxidation of camptothecin and its analogues is central in the semi-synthesis of a wide variety of camptothecin-derived drugs such as irinotecan and topotecan. TDC, tryptophan decarboxylase; CYP450, cytochrome P450 enzyme from *C. acuminata*. The *C. acuminata* artwork was created by K. Davis.

With tremendous abilities to interact with biological targets, specialized metabolites or natural products play a crucial role in plants’ adaptation and have long been exploited to alleviate many illnesses in human. Some of the best-known examples include camptothecin (CPT), vinblastine, and paclitaxel, all of which are potent drugs or promising candidates to be developed further for anticancer treatments^1^. Among these, CPT (**1**, Fig. 1) is a pentacyclic monoterpenoid indole alkaloid (MIA) with a quinoline moiety naturally occurring in *Camptotheca acuminata*, also known as the happy tree in its native habitats in southern China. Despite CPT’s anti-tumour activity, its clinical uses are limited due to its hydrophobicity and adverse side effects^2^. Since 1966^3^, CPT has served as a lead compound and precursor for the synthesis of more clinically useful anticancer drugs, such as 10-hydroxycamptothecin (10HCPT, **2**)^4^, SN-38 (7-ethyl-10HCPT)^5^, irinotecan (7-ethyl-10-[4-(1-piperidino)-1-piperidino]carbonyloxyCPT, or Camptosar^®^) (**3**)^6^ and topotecan (9-[(dimethylamino)methyl]-10HCPT, or Hycamtin^®^) (**4**)^7^, of which irinotecan is listed in the World Health Organization’s Model List of Essential Medicines^8^. These compounds are potent inhibitors of DNA topoisomerase I activity and are widely used to treat several cancers, including lung, cervical, ovarian, colon, uterine, and brain cancers^9–11^. Sales of irinotecan and topotecan have totalled over 15 billion US dollars as of 2018^12^ and the pharmaceutical industry has been relying on 10HCPT for the semi-synthesis of many C-10-modified CPT analogues (Fig. 1).

The clinical potential of plant natural products is often not fully realized because of their rare occurrence in a few slow-growing and/or endangered plant species and at low natural abundance. CPT and derivatives are not the exceptions as *C. acuminata* produce CPT and 10HCPT in minute amounts (<0.0005% dry weight)^13^ in bark and seeds^14^. Moreover, other CPT derivatives such as 11-hydroxycamptothecin (11HCPT, (**5**)), which exhibits a much greater therapeutic index than CPT, occur in even lower quantities^15^, limiting their clinical use. Isolation of CPT from plants, followed by chemical derivatization in harsh conditions, remains the major source for the semi-synthesis of irinotecan, topotecan and other CPT-based chemotherapeutic agents^16–20^. Conventional production strategies present challenges associated with purity and scale-up of the compounds, contributing to high costs and inaccessibility. For instance, the C-H functionalization in topotecan semi-synthesis from CPT involves partial hydrogenation and oxidation with the catalysts Pd/C and Pb(OAc)_4_ to yield 10HCPT (**2**)^21^, followed by condensation with formaldehyde and dimethylamine^22–25^. Although this method can deliver topotecan at feasible yields, it requires labour-intensive processes and the use of toxic reagents on industrial scales^21^.

Although CPT derivatives carry remarkable diversity and complexity, further diversification is essential to expand the chemical space to meet the demand for new drugs with improved therapeutics. The low level of HCPTs in the plant and their challenging semi-synthesis restrict access to, and diversification of, CPT derivatives and their clinical uses. This challenge has prompted a quest for biocatalysts involved in CPT biosynthesis and biotransformation in nature, as, in contrast to the often arduous chemical synthesis, enzyme-based processes can act on complex substrates with high efficiency and regio- and stereo-selectivity at mild conditions (*e.g*., physiological temperature and pH). Here we report the discovery of oxidative enzymes from *C. acuminata* that enable the regio-specific oxidations of CPT to HCPTs. We further demonstrated that these enzymatic products could be readily diversified by chemical means to allow for access to structurally diverse CPT scaffolds that are keys in the production of a series of anticancer agents, including SN-38, topotecan, irinotecan, and ten other potentially valuable CPT analogues.

## Results

### Discovery of CPT oxidative enzymes

Targeted metabolomics studies of *C. acuminata* showed that although CPT accumulates in young leaves, its oxidized derivatives (HCPTs) are primarily found in stems, fruits and bark (Supplementary Fig. 1A). Therefore, we speculated that *C. acuminata*’s genes encoding for enzymes involved in converting CPT to HCPTs would be highly expressed in stems, fruits and bark^26–29^. We focused our search for CPT oxidative enzymes within the cytochrome P450 monooxygenases (CYP450s), as they are the main factors in the oxygenation of plant specialized metabolites^30–34^.

**Fig. 2:**
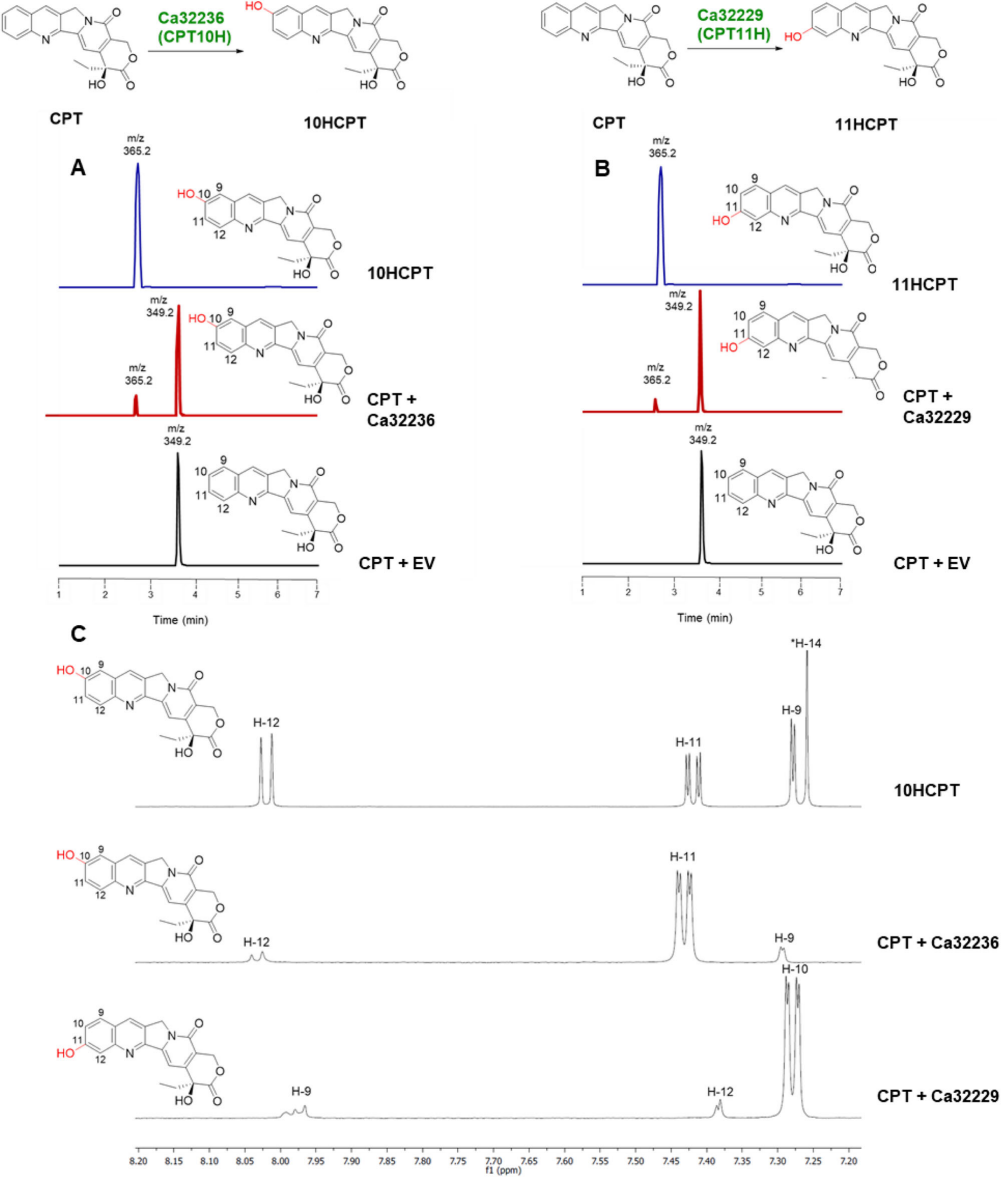
Camptothecin oxidation by Ca32229 and Ca32236. **A**, **B** Extracted ion chromatograms from LC-MS analysis showing the in vivo conversion of CPT to 10HCPT (2.44 min, **A**) and 11HCPT (2.42 min, **B**) by Ca32236 and Ca32229, respectively. **C** NMR spectrum of hydroxylated products with the ^1^H NMR spectrum of 10HCPT standard showing the aromatic protons of ring A and H-14 (top, 7.20–8.20 p.p.m.), and 1D-TOCSY (50 ms spin-lock time) NMR spectra of aromatic protons on ring A of 10HCPT produced by Ca32236 (middle) and of 11HCPT produced by Ca32229 (bottom). *H-14 peak of 10HCPT is not shown in the 1D-TOCSY spectra as there is no correlation between H-14 and aromatic protons of ring A. CPT: camptothecin; HCPT: hydroxy-CPT; EV: empty vector (negative control).

Using the available *C. acuminata* transcriptome and genome data^35,36^ for a self-organizing map analysis^37^ (Supplementary Fig. 1B), we identified nine candidates that show similar expression patterns with those of other MIA biosynthetic genes and 10HCPT accumulation (Supplementary Fig. 1C). These candidates belong to different CYP450 clades (Supplementary Fig. 2A). To test for enzymatic activities, we cloned these CYP450 candidate-coding sequences into the galactose-inducible dual expression vector pESC-Leu2d with a redox partner cytochrome P450 reductase (CPR)^38^. Then, 10 µM CPT was fed to 100 µL cultures of the *Saccharomyces cerevisiae* yeast transformed with the vector for 48 h. Only yeast harbouring pESC-Leu2d::CPR/Ca32236 showed the consumption of CPT and the formation of a new product with a *m*/*z* 365.2, an increase in 16 amu as compared to that of the substrate (*m*/*z* 349.2) and retention time corresponding to 10HCPT as evidenced by liquid chromatography-mass spectrometry (Fig. 2A). No enzymatic product was observed when CPT was incubated with yeast expressing empty vector or any of the other candidates. Similarly, in vitro assays with microsomal fractions of yeast transformed with pESC-Leu2d::CPR/Ca32236 showed that in the presence of NADPH, CPT was consumed and a new product with *m*/*z* 365.2 was formed as evidenced by LC-MS analysis (Supplementary Fig. 3), signifying an oxidation event.

In addition to 10HCPT, *C. acuminata* also produces a limited amount of 11HCPT. Using Ca32236 as a query, we searched for other putative CPT oxidative enzymes in *C. acuminata* transcriptomes and found three homologues, namely Ca32234, Ca32229, and Ca32245, sharing 80%–93% amino acid identity (Supplementary Fig. 2B). Using the same in vivo assay system^39^ (Fig. 1), we found that cultures of yeast harbouring a plasmid with one of these candidates, pESC-Leu2d::CPR/Ca32229, produced a compound with the same *m*/*z* value (365.2) of the 10HCPT derivative but a different retention time in LC-MS analysis (Fig. 2B).

To rigorously confirm the structure of the compounds produced by Ca32229 and Ca32236, the transgenic yeast cultures were upscaled to 1 L. Approximately 5–8 mg of the two products were purified and subjected to ^1^H, ^13^C and one-dimensional total correlation spectroscopy nuclear magnetic resonance (NMR) analyses. The NMR data confirmed that both Ca32236 and Ca32229 catalysed hydroxylations of CPT (Supplementary Figs. 4 and 5). Ca32236 hydroxylated CPT at C-10 to produce 10HCPT (Fig. 2, Supplementary Fig. 4 and Supplementary Note), whereas Ca32229 catalysed the hydroxylation at C-11 to yield 11HCPT (Fig. 2, Supplementary Fig. 5 and Supplementary Note)^39^. Ca32236 and Ca32229 were thus named CPT 10-hydroxylase (CPT10H) and CPT 11-hydroxylase (CPT11H), respectively. NMR data of the substrate CPT was also included for comparison (Supplementary Fig. 5). No other products were detected.

Next, to investigate the substrate scopes of the newly found enzymes, we assayed the two enzymes with 18 alkaloids representing different MIA structural subgroups including β-carbolines, ajmaline, heteroyohimbines, and quinolines (Supplementary Fig. 6). Results showed that the substrate range of CPT10H and CPT11H is restricted to the CPT scaffold. Intriguingly, both CPT10H and CPT11H accepted the commercially available 7-ethylcamptothecin to produce the antineoplastic drug SN-38 (7-ethyl-10HCPT)^40^ (Supplementary Fig. 7A) and its isomer 7-ethyl-11HCPT (Supplementary Figs. 5, 6, and 7B; Supplementary Note), respectively. CPT11H also accepted 10HCPT to produce low amounts (7% conversion) of 10,11-dihydroxyCPT (Supplementary Figs. 6, 7C, and 8, and Supplementary Note). However, 11HCPT was not accepted by CPT10H (Supplementary Fig. 7D). Of note, CPT10H and CPT11H also converted 9-amino-CPT to two new products (Supplementary Figs. 6 and 9). The limited availability of 9-amino-CPT and low conversion rate (9%) precluded the product structure elucidation by NMR spectroscopy. It is speculated that the products are 9-amino-10HCPT and 9-amino-11HCPT (9A10HCPT and 9A11HCPT, respectively; Supplementary Fig. 9) based on the observed *m*/*z* (380.1, an increase in 16 amu as compared to that of the substrate (*m*/*z* 364.1)) and the regio-specificity of CPT10H and CPT11H toward C-10 and C-11, respectively, on the CPT scaffold. Altogether, these enzymes could produce seven products from the CPT scaffold (Supplementary Table 1), of which 11HCPT, 10,11-dihydroxyCPT and putative 9-aminohydroxyCPTs have not been reported in any biosynthetic or synthesis studies, whereas 7-ethyl-11HCPT has been described elsewhere^41,42^.

### Chemoenzymatic synthesis of CPT analogues

**Fig. 3:**
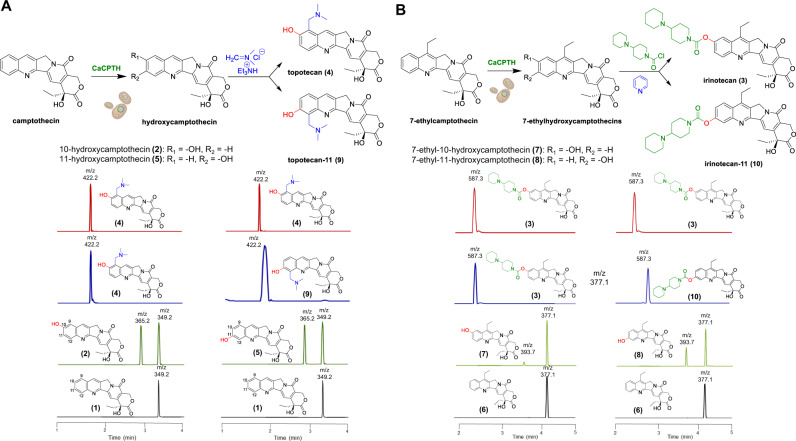
Reaction schemes and LC-MS analysis of the production of camptothecin analogues using camptothecin hydroxylases. **A** Chemoenzymatic synthesis of topotecan (**4**) (Hycamtin^®^) and topotecan-11 (12-[(dimethylamino)methyl]-11-hydroxycamptothecin) (**9**) from camptothecin (**1**). **B** Chemoenzymatic synthesis of irinotecan (**3**) (Camptosar^®^) and irinotecan-11 (7-ethyl-11-[4-(1-piperidino)-1-piperidino]carbonyloxycamptothecin) (**10**) from 7-ethylcamptothecin (**6**). Each LC-MS analysis panel include chromatograms for standard (top, red), chemoenzymatic product (second from top, blue), enzymatic product (second from bottom, green) and starting material (bottom, black).

We envisioned that the key advantage of these oxidative enzymes lies in the opportunity to functionalize the inert C-H bond and to further diversify the products to obtain valuable CPT-based scaffolds. With the newly discovered regioselective CPT hydroxylases, we next demonstrated combinatorial enzymatic and chemical syntheses of CPT analogues topotecan and irinotecan, and their 11HCPT-derived isomers from CPT (Fig. 3). First, we optimized the enzymatic conversion of CPT to HCPTs in yeast expressing CPT hydroxylases. The initial in vivo conversion rate maximized at 10% (Fig. 2), possibly because CPT is insoluble and the native yeast topoisomerase I is sensitive to CPT. We then investigated and optimized different growth conditions and achieved a yield up to 40% from transgenic yeast grown in YPA medium with 2% galactose and 10% glycerol for 48 h. To further increase the yield, we expressed the CPT hydroxylases in SMY75-1.4A yeast strain (Δ*erg6* Δ*top1*), which was previously engineered to allow better penetration of, and improved resistance to, topoisomerase I inhibitors such as CPT^43^. As a result, we obtained a markedly improved conversion of CPT, up to 67% (12 mg/L of 10HCPT and 11 mg/L of 11HCPT from 18 mg/L starting CPT in the crude extract, which yields 9.4 mg/L of pure 10HCPT and 8.1 mg/L of pure 11HCPT after further purification by semiprep HPLC) (Supplementary Table 1). This incredible in vivo enzymatic conversion rate and high regio-selectivity in mild conditions surpassed a typical chemical synthesis reaction (~50%–60%)^21^, affording 10HCPT and 11HCPT for the following chemoenzymatic process (Fig. 3) to produce clinically essential compounds topotecan and irinotecan as well as other derivatives.

Next, treatment of enzymatically produced 10HCPT with an appropriate iminium reagent, *N,N*-dimethylmethyleneiminium chloride, yielded 9-[(dialkylamino)methyl]-10HCPT, commonly known as topotecan (Fig. 3A and Supplementary Fig. 10A)^22^. When the enzymatic product 11HCPT was allowed to react with the same iminium reagent, we obtained total conversion to the new product 12-[(dialkylamino)methyl]-11HCPT (topotecan-11) (Fig. 3A, Supplementary Figs. 10B and 11A, and Supplementary Note). Likewise, using the enzymatic products 7-ethyl-10HCPT and 7-ethyl-11HCPT with [1,4′]bipiperidinyl-1′-carbonyl chloride in pyridine, we achieved conversions to the clinically important drug irinotecan and its 11HCPT-derived isomer, 7-ethyl-11-[4-(1-piperidino)-1-piperidino]carbonyloxyCPT (irinotecan-11) (Fig. 3B, Supplementary Figs. 11B and 12, and Supplementary Note). Furthermore, using a halogenated reagent such as *N-*bromosuccinimide on 10HCPT and 11HCPT derived from in vivo biosynthesis afforded 9-bromo-10HCPT and 12-bromo-11HCPT (Supplementary Figs. 13, 14 and 15, and Supplementary Note). All new chemoenzymatic products were confirmed by LC-MS (Supplementary Figs. 10, 12 and 13), high-resolution MS (Supplementary Note) and NMR analyses (Supplementary Figs. 5, 8, 11, 14 and 15, and Supplementary Note). The formation of topotecan and irinotecan products was also validated on LC/MS and NMR with authentic standards (Fig. 3 and Supplementary Figs. 10A and 12A).

In total, we achieved the biosynthesis and chemoenzymatic production of 13 CPT analogues from CPT (Supplementary Fig. 16). These products encompass compounds naturally occurring in plants (10HCPT and 11HCPT) and clinically active semi-synthetic drugs (SN-38, topotecan and irinotecan). The products include four novel compounds, namely 12-bromo-11HCPT, topotecan-11 (12-[(dimethylamino)methyl]-11HCPT), 10,11-dihydroxyCPT and irinotecan-11 (7-ethyl-11-[4-(1-piperidino)-1-piperidino]carbonyloxyCPT), all of which are not readily accessible either from plants or via conventional chemical C-H functionalization approach. All the chemoenzymatic conversions were completed at room temperature as no substrates, or decomposition products, were detected at the end (Fig. 3 and Supplementary Figs. 10, 12, and 13).

## Discussion

CPT is a powerful but not ideal anticancer drug, owing to its low solubility, undesirable side effects and drug resistance^13^. Chemical substitutions on the CPT scaffold are thus required to improve its potency^44^. Hydroxylations at C-10 and C-11 on ring A of the CPT scaffold are critical features in designing active CPT derivatives^39^, with the semi-synthetic 10HCPT serving as the precursor for the commercial synthesis of the anticancer drugs topotecan and irinotecan^22,25^. Although selective functionalization of unactivated C(*sp*^3^)—H bonds in natural products is especially chemically challenging due to their inherent complexity with various chiral centres and functional groups, natural selection provides elegant enzymatic tools that can help overcome these hurdles. Of these, CYP450s stand out as key and tractable biocatalysts with an ability to activate C-H bonds via oxidation with striking chemo-, regio- and stereo-selectivities. The ability of the newly discovered CYP450-based CPT hydroxylases to oxidize a variety of CPT-derived scaffolds (Supplementary Fig. 6) allowed us to employ a chemoenzymatic pipeline leading to potent anti-tumour CPT derivatives (Fig. 3 and Supplementary Fig. 16). Importantly, the new enzymatic product 11HCPT and its derivatives in this study have been known to exhibit a much greater therapeutic index with less toxicity than CPT^45^, with 11HCPT derivatives such as 7-ethyl-11HCPT overcoming interpatient variability and drug resistance compared with irinotecan^46^.

The rising need for anticancer CPT derivatives requires more sustainable and direct chemoenzymatic steps starting from CPT at mild conditions (pH 7, 30 °C) (Fig. 3) as compared to chemical synthesis. The high regio-selectivity (for the C-10 and C-11 positions) and conversion rate (62%–67%) of CPT hydroxylases afford the production of specific HCPTs and derivatives with chemical decorations at desired positions. Among the new chemoenzymatic products, the bromo-CPT derivatives are of significant note. Halogenated organic compounds are scarce in nature, yet they constitute up to 15% of the pharmaceutical products on the market^47^ and the bromo-HCPTs produced in this work (Supplementary Figs. 13–16) could potentially provide starting handles for selective arylation via cross-coupling^48^ to further diversify the CPT-derived products with new bioactivity potentials.

More than half a century since the isolation of CPT from *C. acuminata* and 40 years after the first report on HCPT chemical synthesis^21,49,50^, the discovery and application of CPT hydroxylases in this study open another window into the largely elusive CPT metabolism. It also represents a greener alternative to chemical semi-synthesis of CPT derivatives and a significant expansion of the CPT chemical space, paving the way for the further regioselective functionalization of the rigid polycyclic alkaloid structures with new bioactive molecules.

## Methods

### Identification and cloning of candidates

Publicly available transcriptomic and metabolomic data of seven different organs of *C. acuminata* (http://medicinalplantgenomics.msu.edu/contacts.shtml) were filtered for contigs with FPKM (fragments per kilobase of exon per million fragments mapped) expression values higher than zero for more than half of the organs (FPKM expression values of zero for more than half of the treatments or with zero expression variance across the samples were removed). Self-organizing maps were applied and visualized in R (RStudio 1.0.136, RStudio, Inc.) with the Kohonen package as reported before^30^. The map was assigned to give about 50 contigs per node. Cytochrome P450 candidates in the same nodes or neighbouring nodes with similar expression patterns with previously reported genes were selected for cloning and testing for activity. Nine CYP450 candidates belonging to different CYP450 families, including CYP71, CYP72, CYP76, CYP81 and CYP82, were identified. The full-length coding regions of CYP450s candidates were amplified using cDNA derived from total RNA of *C. acuminata* stems and leaves using Platinium^TM^ SuperFi^TM^ PCR Mastermix (Thermofisher) with appropriate primers (Supplementary Table 2). As Ca32229, Ca32236 and Ca32245 share very high sequence identity (Supplementary Fig. 2), especially at the N terminus, we had these genes synthesized by Twist BioSciences (CA, USA) based on the available transcriptome^35^.

### Protein expression

For heterologous expression of Flag-tagged CYP450s in yeast (*S. cerevisiae*), the full-length coding region of each CYP450 candidate was cloned between SpeI and NcoI restriction sites of MCS1 of the dual plasmid *pESC-Leu2d* with a CPR in MCS2^30,38^ yielding pESC-Leu2d::CYP/CPR using In-Fusion cloning system (Takara Clontech). The resulted pESC-Leu2d::CYP/CPR was transformed to the protease-deficient yeast strain YPL 154C:Pep4KO and yeast harbouring pESC-Leu2d::CPR was used as the negative control. To optimize HCPT production, we used Δerg6 Δtop1 yeast double mutant strain SMY75-1.4A43, which was previously generated to allow better penetration of, and improved resistance to, topoisomerase I inhibitors such as CPT. The conditions for yeast culture, microsome preparation and immunoblot analysis are included in the Supplementary Methods.

#### Enzyme assays

For screening in vivo CPT oxidation activities, 10 µM CPT was fed to 100 µL cultures of YPLC 154C:Pep4KO yeast transformed with the vector for 48 h. The culture volume can be scaled up to 2 L with the CPT concentration up to 50 µM, to produce sufficient products for structural characterization and/or semi-synthesis of CPT derivatives (Supplementary Information). Standard in vitro assays were performed at 30 °C for 1 h in 100 μL of 100 mM HEPES-NaOH (pH 7.5) containing 10 mg of total microsomal proteins, 50 μM substrate (Supplementary Fig. 3) and 250 μM NADPH on a gyratory shaker with agitation (750 r.p.m.). Reactions were stopped by adding 800 μL methanol. The reaction mixture was extracted twice with methanol to precipitate and remove proteins. The supernatant was subjected to LC-MS/MS analysis (Supplementary Methods).

## Supplementary information

Supplementary Materials

## Data Availability

The authors declare that all data supporting the findings of this study, including genetic sequence accession codes and web links for publicly available datasets, are available within the paper and the Supplementary Information.
