# Supplementary material for: Discovering and harnessing oxidative enzymes for chemoenzymatic synthesis and diversification of anticancer camptothecin analogues
Source: Commun Chem. 2021 Dec 16;4:177. doi: 10.1038/s42004-021-00602-2 (PMC9814082; doi:10.1038/s42004-021-00602-2)
Supplement: Supplementary file 1 — Supplementary Materials [file 42004_2021_602_MOESM1_ESM.pdf]

Supplementary Information

Discovering and harnessing oxidative enzymes for chemoenzymatic synthesis and diversification of anticancer camptothecin analogues

Tuan-Anh M. Nguyen[1†], Trinh-Don Nguyen[1†], Yuen Yee Leung[1], Matthew McConnachie[1], Oleg Sannikov[2], Zhicheng Xia[2], Thu-Thuy T. Dang[1*]

[1]Department of Chemistry, Irving K. Barber Faculty of Science, University of British Columbia
3427 University Way, Kelowna, BC V1V 1V7, Canada

[2]Department of Chemistry, Faculty of Science, University of British Columbia
2036 Main Mall, Vancouver, BC V6T 1Z1, Canada

[†]These authors contribute equally to this work.

*Corresponding author: **thuy.dang@ubc.ca**

Table of Contents

Supplementary methods **3**

Supplementary note **6**

Supplementary Figure 1. Identification of CPT oxidative enzyme candidates 7

Supplementary Figure 2. Sequence analysis of CYP450 candidates and alignment of Ca32229, Ca32245 and Ca32236 8

Supplementary Figure 3. Protein expression and *in vitro* assays of CYP450s 9

Supplementary Figure 4. ^{1}H NMR and ^{13}C NMR spectra of products from *in vivo* assay of CaCYP32236/CPR 10–11

Supplementary Figure 5. ^{1}H NMR and ^{13}C NMR spectra of products from *in vivo* assay of Ca32229/CPR 12–13

Supplementary Figure 6. Substrate specificity of CPT hydroxylases, Ca32236 (CPT 10-hydroxylase) and Ca32229 (CPT 11-hydroxylase)...... 14

Supplementary Figure 7. Oxidation of 7-ethyl-CPT by Ca32229 and Ca32236 15

Supplementary Figure 8. ^{1}H NMR spectrum of products from *in vivo* assay of Ca32229/CPR with 10HCPT as substrate producing 10,11-dihydroxyCPT 16

Supplementary Figure 9. Oxidation of 9-amino-CPT by Ca32229 producing 9-amino-11HCPT, and Ca32236 to produce 9-amino-10HCPT 17

Supplementary Figure 10. Chemoenzymatic production of topotecan and topotecan-11 (12-[(dimethylamino)methyl]-11HCPT) 18

Supplementary Figure 11. ^{1}H NMR and ^{13}C NMR spectra of chemoenzymatic reaction products topotecan-11 (12-[(dimethylamino)methyl]-11HCPT) (A) and irinotecan-11 19–20

Supplementary Figure 12. Chemoenzymatic production of irinotecan (A) and irinotecan-11 21

Supplementary Figure 13. Chemoenzymatic production of brominated HCPTs using CPT 10-hydroxylase (A) and CPT 11-hydroxylase (B) as biocatalysts 22

Supplementary Figure 14. ^{1}H NMR and ^{13}C NMR spectra of bromination reaction of 10HCPT as substrate producing 9-bromo-10HCPT and 11HCPT as substrate producing 12-bromo-11HCPT. 23–24

Supplementary Figure 15. 1D-TOCSY NMR spectra of brominated products of 10HCPT and 11HCPT 25

Supplementary Figure 16. Summary of CPT analogs produced by chemoenzymatic reactions of camptothecin 26

Supplementary Table 1. Summary of yeast *in vivo* assay yields of CPT hydroxylases with CPT-scaffold substrates 27

Supplementary Table 2. Primers used to construct pESC-Leu2d expression vectors 28

Supplementary references **29**

SUPPLEMENTARY METHODS

Plants and chemicals

Camptotheca acuminata cuttings were obtained from Quarryhill Botanical Garden (California, USA) and the Huntington Library, Art Collections, and Botanical Gardens (California, USA). The cuttings were snap-frozen upon receipt for RNA isolation. Secologanin, ajmaline, tetrahydroalstonine, serpentine, and yohimbine were purchased from Northernchem Inc. (Ontario, Canada). All other chemicals were of analytical grade from Sigma-Aldrich.

Phylogenetic analysis

Unrooted neighbour-joining phylogenetic tree for CYP450 candidates from this study and other reported CYP450s from other organisms were performed using the Geneious Tree Builder program in the Geneious software package (Biomatters). The names, abbreviations and GenBank accession numbers of the included sequences are: *C. acuminata* CPT 10-hydroxylase, CaCPT10H, OK631678; *C. acuminata* CPT 11-hydroxylase, CaCPT11H, OK631675; *C. acuminata* Ca32245, MN631049; *Arabidopsis thaliana* CYP81D1, AtCTP81D1, NP_568533.2; *A. thaliana* CYP81F1, AtCTP81F1, O65790.2; *A. thaliana* CYP81H1, AtCTP81H1, NC_003075.7; *A. thaliana* CYP81K1, AtCTP81K1, NC_003076.8; *Catharanthus roseus* alstonine synthase, CrCYP71AY1, KF309243.1; *C. roseus* tabersonine 16-hydroxylase, CrCYP71D12, FJ647194.1; *C. roseus* geraniol 10-hydroxylase, CrG10H, Q8VWZ7.1; *C. roseus* 7-deoxyloganic acid 7-hydroxylase, Cr7DLH (CYP72A224), AGX93062.1; *C. roseus* CYP71BT1, AHK60840.1; *C. roseus* secologanin synthase, CrSLS, Q05047; *C. roseus* tabersonine 19-hydroxylasem, CrCYP71BJ1 (T19H), ADZ48681; *C. roseus* geissoschizine oxidase, CrCYP71D1V1, JN613015.1; *C. roseus* tabersonine 16-hydroxylase, ACM92061; *C. roseus* tabersonine 6,7-epoxidase, CrCYP71D521, AVH80640; *Camellia sinensis* CYP81D11, XP_028101205.1; *Echinochloa phyllopogon* CYP81A12, BAO73908.1; *Hypericum calycinum* CYP81AA1, ANC33509.1; *Rauwolfia serpentine* sarpagan bridge enzyme, RsSBE, P0DO13.1; *Sesamum alatum* CYP81Q3, BAE48236.1; *Papaver somniferum* CYP82X1, AFB74614.1; *P. somniferum* CYP82Y1 AFB74617.1; *P. somniferum* CYP82X2, AFB74617.1; *Sesamum indicum* CYP81E8, NP_001306620.1; *Salvia miltiorrhiza* CYP82V2, KP337709.1L; *Sesamum radiatum* CYP81Q2, AB194715.1; *Theobroma cacao* CYP71D9, XM_018120397.1; and *Tabernanthe iboga* ibogamine 10-hydroxylase (I10H), TiCYP76, MH454074.1.

Yeast culture, microsome preparation and immunoblot analysis

For routine yeast culture, the transgenic yeast strain was inoculated in 2 mL of synthetic complete (SC) medium lacking leucine (SC-Leu) containing 2% (w/v) glucose and cultured overnight at 30 °C and 250 rpm. The culture was subsequently diluted 100-fold to an OD_{600} of 0.05 in SC-Leu supplemented with 2% (w/v) glucose and cultured for 16 hr. Yeast was then harvested and sub-cultured for 24 hr in YPA medium containing 2% (w/v) galactose to induce the production of recombinant CYP450s. Yeast cells were harvested by centrifugation and lysed for 2 min using a micro-bead beater (VWR) and 500-µm diameter glass beads in TES (0.6 M sorbitol in TE) buffer. The resulting lysate was subsequently centrifuged at 10,000 g for 15 min at 4 °C. The supernatant was then transferred to a new tube and centrifuged at 40,000 *g* for 60 min at 4 °C. Finally, the pellet containing microsomes was resuspended with TEG buffer (20% (v/v) glycerol in TE). Expression of Ca32229 and Ca32236 was confirmed by immunoblot analysis of microsomal fractions prepared from *S. cerevisiae* cultures harbouring the *pESC-Leu2d::CPR/Ca32229* and *pESC-Leu2d::CPR/Ca32236* vectors using α-FLAG M2 antibodies (ThermoFisher Scientific) detectable with SuperSignal West Pico Chemiluminescent Substrate (ThermoFisher Scientific) to probe epitope-tagged recombinant proteins (Supplementary Figure 3).

LC-MS/MS analysis

Enzyme assays were analyzed by ultra-performance liquid chromatography (UPLC) on a Xevo TQ-S Cronos Triple Quadrupole Mass Spectrometry (Waters). For all studies, chromatography was performed on an XBridge BEH XP (10 × 2.1 mm, 1.7 µm) column at a flow rate of 0.6 $mL.min^{-1}$. The column was equilibrated in solvent A (0.1% formic acid) and the following elution conditions were used: 0 min, 5% B (100% acetonitrile); from 0 to 3.5 min, 35% B; from 3.5 min to 3.75 min, 100%B; 3.75 min to 4.75, 100%B; 4.75 to 6 min, 5% B to re-equilibrate the column. Data were analyzed with MassLynx and TargetLynx (Waters)

For high-resolution MS (HRMS) analysis, new compounds were subjected to the Agilent 1290 Infinity system connected to the Agilent 6530 Quadrupole Time-of-Flight (QTOF). Chromatography was performed on an XBridge BEH XP (10 x 2.1 mm, 1.7 µm) column at a flow rate of 0.6 $mL.min^{-1}$. The column was equilibrated in solvent A (0.1% formic acid) and the following elution conditions were used: 0 min, 5% B (100% acetonitrile); from 0 to 3.5 min, 35% B; from 3.5 min to 3.75 min, 100%B; 3.75 min to 4.75, 100%B; 4.75 to 6 min, 5% B to re-equilibrate the column. Data were analyzed with Mass Hunter (Agilent Technologies)

Conversion rate and yield calculation

A calibration curve using camptothecin from 0–50 nM was made for quantification. Peaks areas of LC-MS chromatograms were calculated using MassLynx and TargetLynx from Waters and normalized. The amount of substrate consumption, product formation, conversion, and total product yield was quantified using corresponding calibration curves.

Semi-preparative HPLC and NMR analyses for structure elucidation

A scaled-up yeast in vivo assay with CPT and 7-ethyl-CPT substrates were performed to produce sufficient product quantities of HCPTs and 7-ethyl-HCPT for NMR analysis. The supernatant of the assays was obtained by centrifugation. The crude containing HCPT and 7-ethyl-HCPT in the supernatant were collected by liquid-liquid extraction with ethyl acetate and chloroform, respectively. Product purification from the concentrated sample was performed by a semi-preparative HPLC system with Kinetex® 5 µm EVO C18 100 Å, 10 x 250 mm column at a flow rate of 1.5 mL.min^{-1}. The column was equilibrated in solvent A (water, 0.1 % formic acid) and solvent B (0.1% formic acid in acetonitrile). Then, the following elution conditions were used: 0 min, 10 % B; from 0 to 5 min, 20 % B; from 5 to 25 min, 70 % B; from 25 to 27 min, 90 % B; from 27 to 30 min, 90 % B; from 30 to 31 min; 10 % B; from 31 to 34 min, 10 % B to re-equilibrate the column. Approximately 1 mg of each product was independently dissolved in 600 µL DMSO-d_6 and subjected to ^{1}H NMR analysis on Bruker Avance 600 NMR spectrometer. 1D-TOCSY NMR technique (50 ms spin-lock time) were used afterwards to analyze the overlapped aromatic protons signals with irradiation frequency set at 8.02 ppm. The ^{1}H NMR spectra were analyzed and compared with those of standards and literature for known compounds.

Scale-up and purification of new compounds for chemoenzymatic synthesis of hydroxycamptothecin derivatives

To generate sufficient amounts of HCPTs (10 and 11HCPT) and 7-ethyl-HCPT (7-ethyl-10 and 11HCPT) for the synthesis of topotecan, irinotecan and other compounds, we scaled up the enzymatic reactions. The transgenic yeast strain was inoculated in 2 mL of synthetic complete medium lacking leucine (SC-Leu) containing 2% (w/v) glucose and cultured overnight at 30°C and 275 rpm. The culture was subsequently diluted to an OD_{600} of 0.05 in SC-Leu supplemented with 2% (w/v) glucose and cultured for 16 hr. The yeast was then harvested and sub-cultured for 48 hr in YPA medium containing 2% (w/v) galactose, and 10% glycerol to induce the production of recombinant CYP450s. CPT or 7-ethyl-CPT substrate was fed directly into the culture to reach a final concentration of 50 µM as soon as the yeast was switched from SC-Leu to YPA medium. After 48-hr inoculation, a conversion rate of approximately 70% from CPT or 7-ethyl-CPT to its hydroxylated product was obtained and confirmed by LCMS analysis. The supernatant was collected by centrifugation at 4000 rpm, for 5 minutes. HCPT and 7-ethyl-HCPT were extracted out of reaction matrix by liquid-liquid extraction with ethyl acetate and chloroform, respectively. The solvent was removed by using a rotary evaporator to obtain crude HCPT and 7-ethyl-HCPT substrates for chemical synthesis to topotecan and irinotecan. HCPT and 7-ethyl-HCPT were purified by semi-preparative HPLC prior to the synthesis of derivatives.

Semi-synthesis of topotecan and topotecan-11 (12-[(dimethylamino)methyl]-11HCPT)

Fifteen mg of solid *N,N*-dimethylmethyleneiminium chloride was added into an empty 4 mL reaction flask. Six mg of HCPT substrates from the enzymatic reaction was dissolved by 1 mL isopropanol:chloroform (1:1) and transferred into the reaction flask. Two µL triethylamine was added into the mixture then the reaction mixture was magnetically stirred at room temperature for 24 hr. Then, the mixture was acidified to pH 3–4 with 1 N HCl[1]. The reaction mixture was analyzed by LC-MS/MS method to identify the topotecan product. The solvent in the reaction mixture was removed to dryness *in vacuo*. The dried reaction mixture was dissolved in methanol and the final product was purified by semi-prep HPLC to yield approximately 4 mg dried product. The dried product was dissolved in DMSO-d_6 and subjected to ^{1}H NMR analysis on Bruker Avance 600 NMR spectrometer in order to elucidate the structure of the final product.

Semi-synthesis of irinotecan and irinotecan-11 (7-ethyl-11-[4-(1-piperidino)-1-piperidino]carbonyloxyCPT)

Six mg of solid 4-piperidinopiperidine-1-carbonyl chloride was added into an empty 4 mL reaction flask. One mg of 7-ethyl-HCPT substrates from the enzymatic reaction was dissolved by 200 µL pyridine and transferred into the reaction flask. The reaction mixture was magnetically stirred at room temperature for 2 hr. The reaction mixture was analyzed by LC-MS/MS method to detect the irinotecan product. Pyridine was removed by rotatory evaporator after 2 hr. The dried crude mixture was dissolved in 300 µL water. Then 1.5 mL dichloromethane was used to extract the irinotecan product out of the mixture. Dichloromethane layer was dried *in vacuo* to obtain 1.5 mg dried

product. The dried product was dissolved in DMSO-d_6 and subjected to ^1H NMR analysis on a Bruker Avance 600 NMR spectrometer in order to elucidate the structure of the final product.

Semi-synthesis of brominated HCPTs

An amount of 15 mg of solid *N*-bromosuccinimide (NBS) was added into an empty 4 mL reaction flask. 3 mgs of dried HCPT substrates from the enzymatic reaction were dissolved by 200 µL DMSO (pre-cooled at 4 °C). After that, the substrate was transferred into the flask containing *N*-bromosuccinimide on ice. The mixture was magnetically stirred at room temperature in the dark for 2 hr. The reaction progress was analyzed by LC-MS/MS method to detect the brominated HCPT product. Then, the reaction mixture was transferred into 5 mL cold water, the pH of the mixture was adjusted to 3–4 with 1 N HCl[2]. Water and organic solvent were removed by GeneVac evaporator with a temperature below 40 °C. The dried reaction mixture was then dissolved in methanol, and the pure brominated product was purified by semi-prep HPLC to obtain 1.1 mg dried product. The dried product was dissolved in DMSO-d_6 and subjected to ^1H NMR analysis on a Bruker Avance 600 NMR spectrometer to determine the position of the bromine substituent position.

SUPPLEMENTARY NOTE

The chemical identities of compounds reported in this paper were obtained through spectroscopic and spectrometric analyses.

10-hydroxycamptothecin: *^{1}H-NMR* (600 MHz, DMSO-d$_6$) δ = 10.37 (s, 1H), 8.45 (s, 1H), 8.02 (d, *J* = 9.0 Hz, 1H), 7.42 (dd, *J* = 9.0, 3.0 Hz, 1H), 7.28 (d, *J* = 3.0 Hz, 1H), 7.26 (s, 1H), 6.51 (s, 1H), 5.41 (s, 2H), 5.23 (s, 2H), 1.86 (m, 2H), 0.87 (t, *J* = 7.2 Hz, 3H). *^{13}C-NMR* (150 MHz, DMSO-d$_6$) δ = 173.06, 157.42, 157.11, 150.64, 149.86, 146.38, 143.67, 131.10, 130.39, 130.17, 129.84, 123.57, 118.59, 109.30, 96.51, 72.90, 65.69, 50.64, 30.71, 8.20. HRMS calculated for $C_{20}H_{16}N_2O_5$, 364.1059; found, 364.1075.
11-hydroxycamptothecin: *^{1}H-NMR* (600 MHz, DMSO-d$_6$) δ = 10.44 (s, 1H), 8.54 (s, 1H), 7.97 (d, *J* = 7.8 Hz, 1H), 7.38 (d, *J* = 3.0 Hz, 1H), 7.30 (s), 7.27 (dd, *J* = 8.4, 2.4 Hz, 1H), 6.50 (s, 1H), 5.42 (s, 2H), 5.22 (s, 2H), 1.86 (m, 2H), 0.88 (t, *J* = 7.2 Hz, 3H). *^{13}C-NMR* (150 MHz, DMSO-d$_6$) δ = 172.99, 159.79, 157.35, 152.81, 150.46, 146.35, 139.99, 138.61, 131.73, 130.21, 129.86, 121.05, 119.10, 110.33, 96.89, 72.87, 65.73, 50.59, 30.74, 8.24. HRMS calculated for $C_{20}H_{16}N_2O_5$, 364.1059; found, 364.1070.
10,11-dihydroxycamptothecin: *^{1}H-NMR* (600 MHz, DMSO-d$_6$) δ = 10.35 (s, 1H), 10.15 (s, 1H), 8.34 (s, 1H), 7.37 (s, 1H), 7.28 (s, 1H), 7.26 (s, 1H), 6.46 (s, 1H), 5.40 (s, 2H), 5.18 (s, 2H), 1.88 (m, 2H), 0.88 (m, 3H).
7-ethyl-10-hydroxycamptothecin: *^{1}H-NMR* (600 MHz, DMSO-d$_6$) δ = 10.30 (s, 1H), 8.02 (d, *J* = 9.0 Hz, 1H), 7.40 (m, 2H), 7.24 (s, 1H), 6.49 (s, 1H), 5.41 (d, *J* = 2.4 Hz, 2H), 5.26 (s, 2H), 3.07 (m, 2H), 1.85 (m, 2H), 1.29 (t, *J* = 7.8 Hz, 3H), 0.88 (t, *J* = 7.2 Hz, 3H). *^{13}C-NMR* (150 MHz, DMSO-d$_6$) δ = 172.56, 156.85, 156.72, 150.06, 148.84, 146.43, 142.73, 131.55, 128.18, 128.00, 122.37, 117.99, 104.76, 95.78, 72.40, 69.77, 65.24, 49.45, 30.21, 22.29, 13.36, 7.76. HRMS calculated for $C_{22}H_{20}N_2O_5$, 392.1372; found, 392.1370.
7-ethyl-11-hydroxycamptothecin: *^{1}H-NMR* (600 MHz, DMSO-d$_6$) δ = 10.39 (s, 1H), 8.14 (d, *J* = 9.0 Hz, 1H), 7.38 (d, *J* = 2.4 Hz, 1H), 7.29 (dd, *J* = 9.0, 2.4 Hz, 1H), 7.21 (s, 1H), 6.52 (s, 1H), 5.43 (s, 2H), 5.27 (s, 2H), 3.24 (m, 2H), 1.88 (m, 2H), 1.30 (t, *J* = 7.8 Hz, 3H), 0.88 (t, *J* = 7.2 Hz, 3H). *^{13}C-NMR* (150 MHz, DMSO-d$_6$) δ = 172.55, 156.83, 156.16, 150.64, 149.98, 146.37, 145.36, 129.04, 128.10, 125.28, 120.86, 120.16, 110.68, 96.35, 72.39, 69.77, 65.26, 49.31, 30.27, 22.22, 14.03, 7.75. HRMS calculated for $C_{22}H_{20}N_2O_5$, 392.1372; found, 392.1383.
Topotecan-11: *^{1}H-NMR* (600 MHz, DMSO-d$_6$) δ = 8.65 (s, 1H), 8.12 (d, *J* = 9.0 Hz, 1H), 7.64 (d, *J* = 9.0 Hz, 1H), 7.48 (s, 1H), 5.44 (s, 2H), 5.27 (s, 2H), 4.59 (s, 2H), 2.85 (s, 6H), 1.89 (m, 2H), 0.89 (t, *J* = 7.2 Hz, 3H). *^{13}C-NMR* (150 MHz, DMSO-d$_6$) δ = 172.42, 159.39, 156.87, 152.33, 150.12, 148.51, 145.70, 132.30, 131.41, 127.40, 122.52, 120.03, 119.03, 109.46, 97.29, 80.32, 72.60, 65.46, 63.02, 61.09, 50.21, 30.77, 8.02. HRMS calculated for $C_{23}H_{23}N_3O_5$, 421.1638; found, 421.1643.
Irinotecan-11: *^{1}H-NMR* (600 MHz, DMSO-d$_6$) δ = 8.31 (d, *J* = 9.6 Hz, 1H), 7.88 (d, *J* = 2.4 Hz, 1H), 7.56 (dd, *J* = 9.0, 2.4 Hz, 1H), 7.32 (s, 1H), 6.52 (s, 1H), 5.44 (s, 2H), 5.34 (s, 2H), 3.24 (m, 3H), 1.86 (m, 2H), 1.32 (t, *J* = 7.8 Hz, 3H), 0.88 (t, *J* = 7.2 Hz, 3H), 1.23–4.08 (19H). *^{13}C-NMR* (150 MHz, DMSO-d$_6$) δ = 172.53, 156.77, 152.67, 152.41, 149.95, 145.94, 145.60, 127.85, 125.16, 124.29, 123.45, 120.29, 119.10, 108.08, 96.76, 72.41, 65.29, 62.21, 61.75, 61.56, 52.31, 49.55, 49.43, 45.75, 43.38, 42.85, 30.29, 26.90, 25.29, 22.35, 20.75, 14.04, 7.79. HRMS calculated for $C_{33}H_{38}N_4O_6$, 586.2791; found, 586.2814.
9-bromo-10-hydroxycamptothecin: *^{1}H-NMR* (600 MHz, DMSO-d$_6$) δ = 11.18 (s, 1H), 8.74 (s, 1H), 8.08 (d, *J* = 9.0 Hz, 1H), 7.63 (d, *J* = 9.0 Hz, 1H), 7.29 (s, 1H), 5.42 (s, 2H), 5.30 (s, 2H), 1.86 (m, 2H), 0.88 (m, 3H). ^{13}C-NMR (150 MHz, DMSO-d$_6$) δ = 172.69, 157.06, 154.00, 150.26, 150.16, 145.52, 143.91, 131.62, 130.25, 128.99, 128.74, 122.47, 118.80, 103.95, 96.62, 75.26, 65.40, 50.71, 30.45, 7.90. HRMS calculated for $C_{20}H_{15}BrN_2O_5$, 442.0164; found, 442.0159.
12-bromo-11-hydroxycamptothecin: *^{1}H-NMR* (600 MHz, DMSO-d$_6$) δ = 11.05 (s, 1H), 8.62 (s, 1H), 8.00 (d, *J* = 9.0 Hz, 1H), 7.46 (d, *J* = 9.0 Hz, 1H), 7.36 (s, 1H), 5.44 (s, 2H), 5.27 (s, 2H), 4.73 (s, 1H), 1.87 (m, 2H), 0.89 (m, 3H). *^{13}C-NMR* (150 MHz, DMSO-d$_6$) δ = 172.70, 157.04, 156.79, 153.11, 150.30, 146.91,, 142.07, 132.23, 128.80, 128.53, 127.81, 123.81, 119.16, 106.28, 96.99, 72.70. 65.50, 50.30, 30.52, 8.06. HRMS calculated for $C_{20}H_{15}BrN_2O_5$, 442.0164; found, 442.0159.

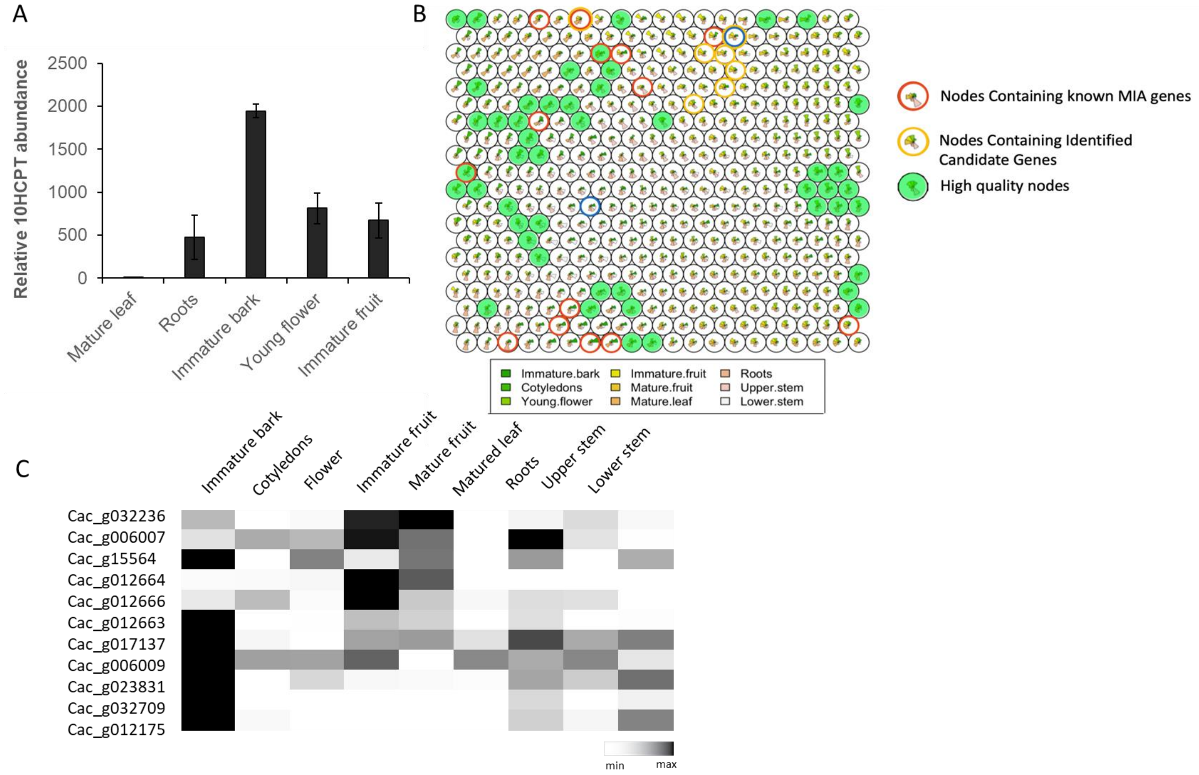

Supplementary Figure 1. Identification of CPT oxidative enzyme candidates. **A**, Abundance of CPT and 10-HCPT in different *C. acuminata* organs with error bars representing standard deviations (*n = 3*). **B**. Self-organizing map code plot showing the nodes from where candidate genes were picked. **C**. Relative abundance of CYP450 candidates in different *C. acuminata* organs (colour scale: white to black shades correspond to low to high abundance levels). Gene expression data are available from http://medicinalplantgenomics.msu.edu/contacts.shtml.

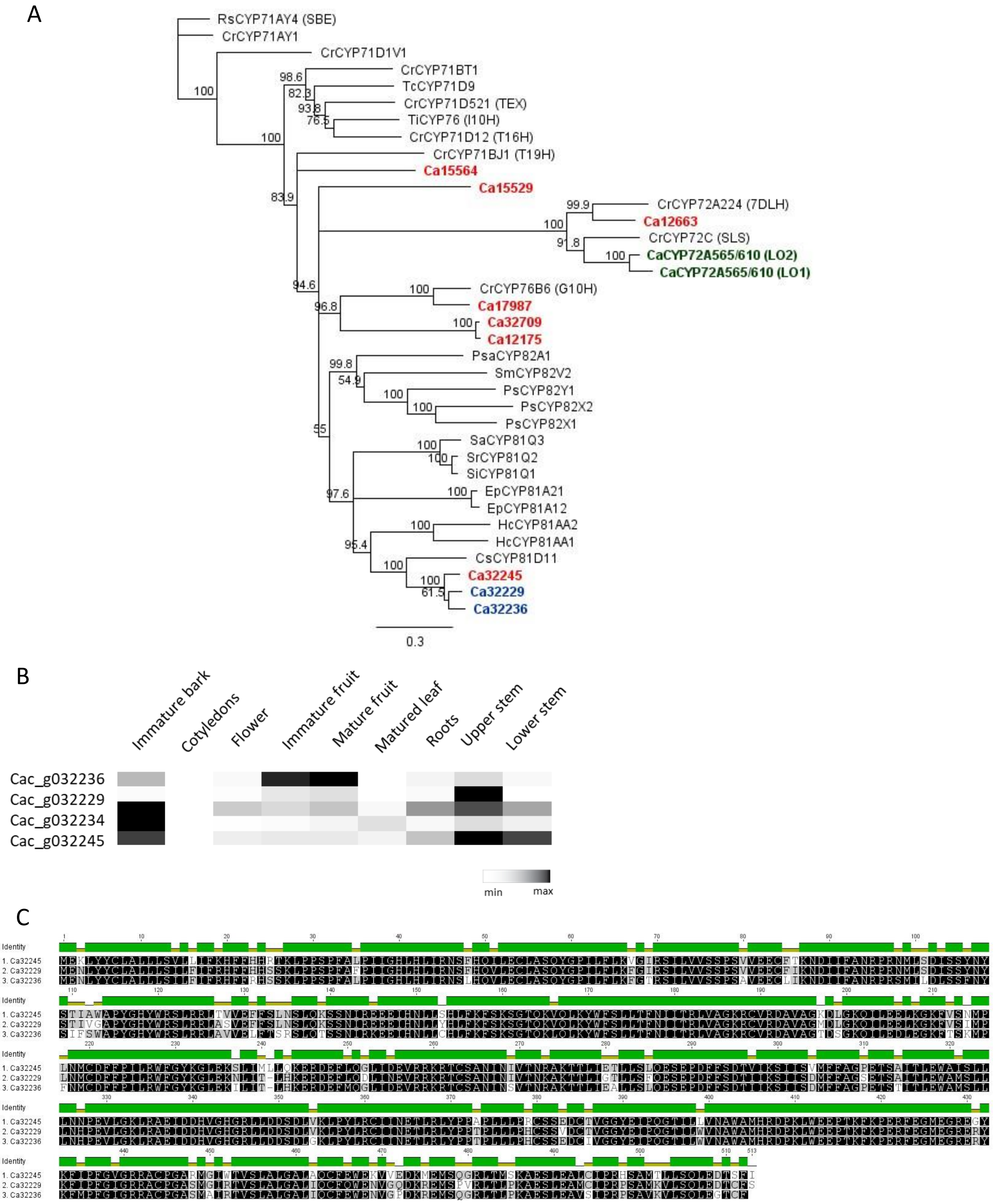

Supplementary Figure 2. Sequence analysis of CYP450 candidates**. A**. Unrooted neighbour-joining phylogenetic tree for CYP450 candidates from this study (red and blue) and previously reported CYP450s from *C. acuminata* and other organisms. Active CPT hydroxylases are shown in blue. Bootstrap frequencies for each clade were based on 1000 iterations. Abbreviations and GenBank accession numbers for each protein are provided in the Material and Methods. B. Relative abundance of Ca32236 homologues in different organs. C. Alignment of Ca32229, Ca32245 and Ca32236. Only Ca32236 and Ca32229 are able to convert CPT to 10HCPT and 11HCPT, respectively, while Ca32245 could not despite its high sequence similarity to the other two.

A

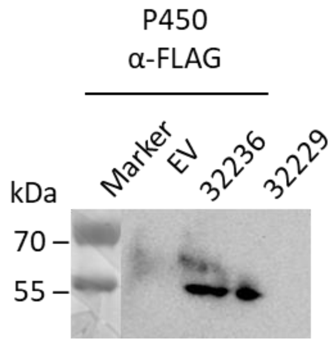

B

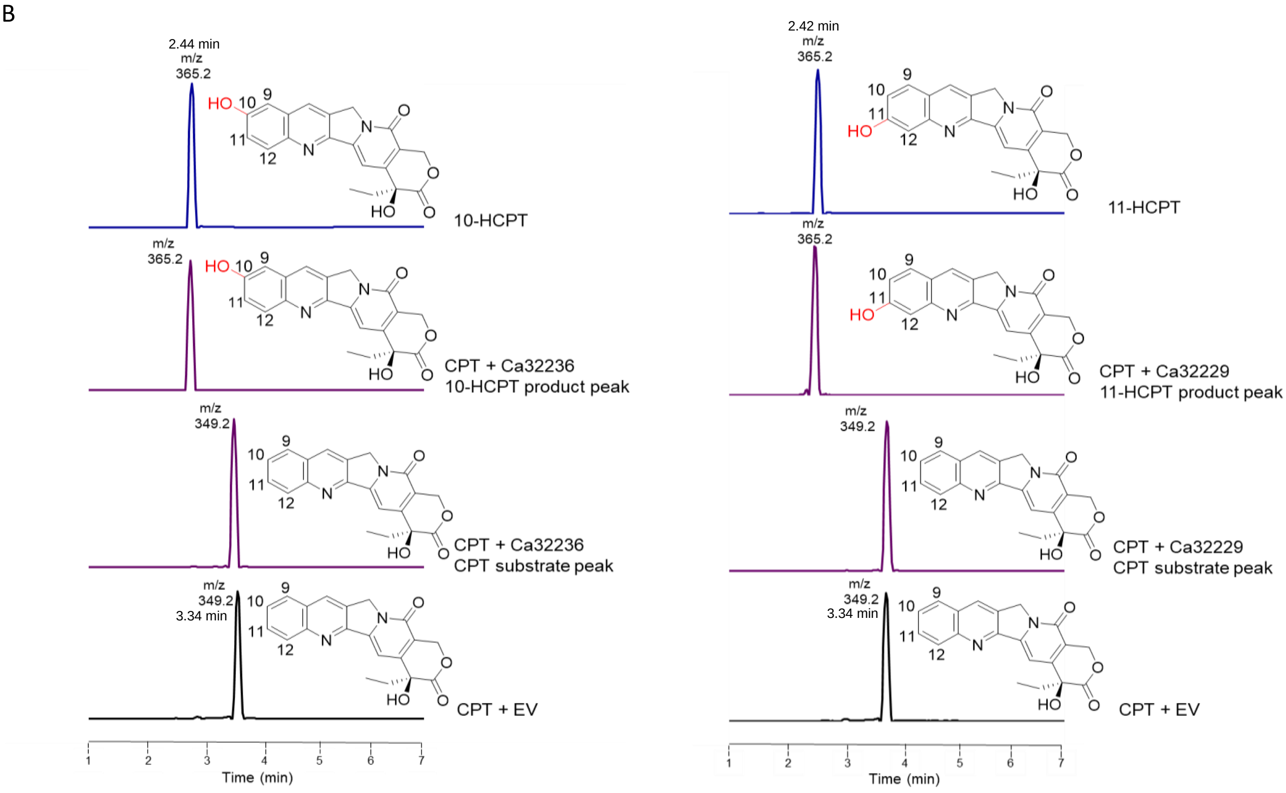

Supplementary Figure 3. Protein expression and *in vitro* assays of CYP450s. **A**. Western blot showing the expression of Ca32229 and 32236 in *Saccharomyces cerevisiae* harbouring pESC-Leu2d::CPR (EV: empty vector), pESC-Leu2d::32229/CPR and pESC-Leu2d::32236/CPR. Protein expression was induced by adding galactose. Recombinant P450 proteins were detected using α-FLAG antibodies. **B**. *In vitro* assays of total microsomal protein extracts of *S. cerevisiae* harbouring Ca32236 (left) and Ca32229 (right) with CPT.

A

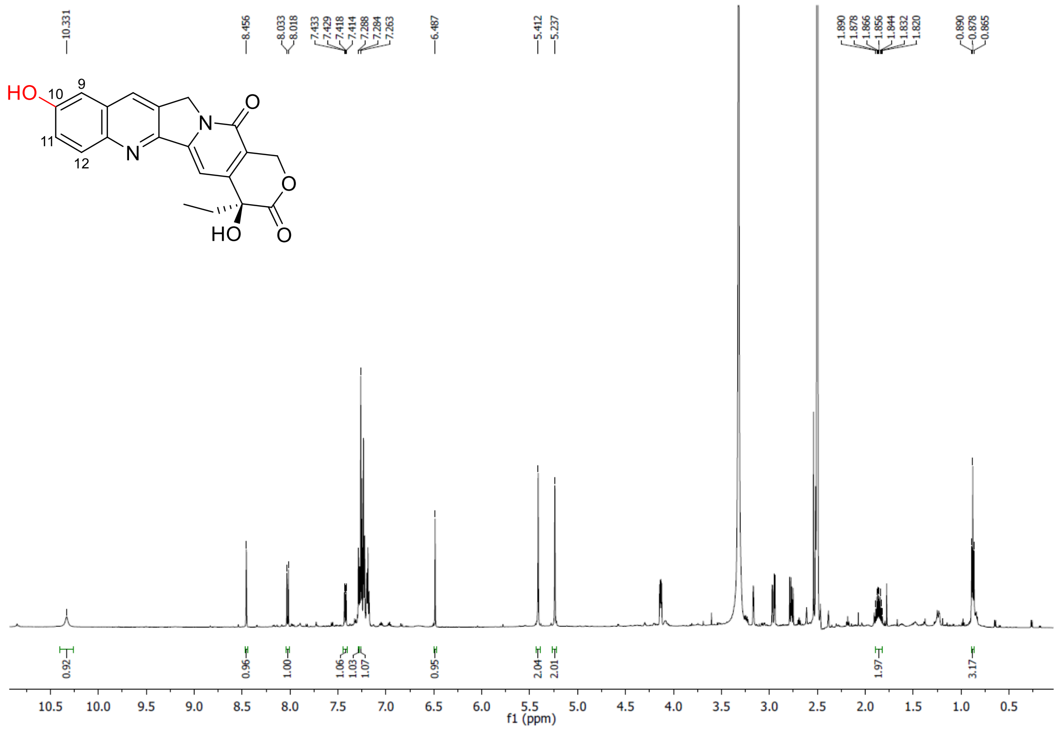
10.331
8.456
8.033
8.018
7.433
7.429
7.418
7.414
7.288
7.284
7.263
6.487
5.412
5.237
1.890
1.878
1.866
1.856
1.844
1.832
1.820
0.890
0.878
0.865
HO
10
9
11
12
N
N
O
O
O
HO
0.92
0.96
1.00
1.06
1.03
1.07
0.95
2.04
2.01
1.97
3.17
f1 (ppm)

B

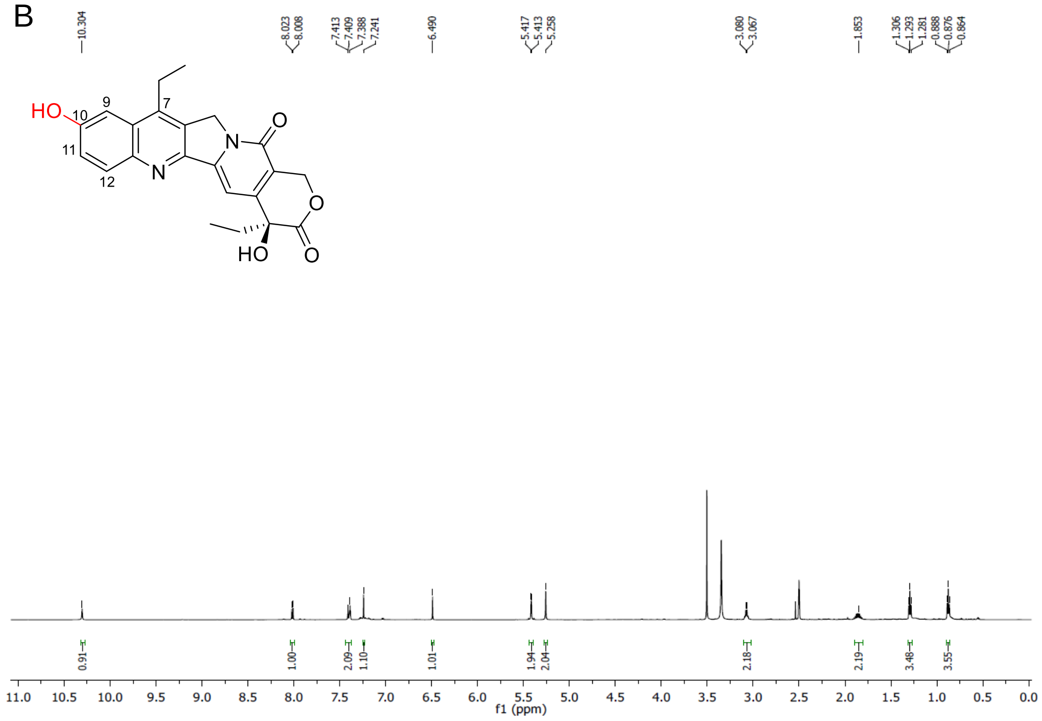
10.304
8.023
8.008
7.413
7.409
7.388
7.241
6.490
5.417
5.413
5.258
3.080
3.067
1.853
1.306
1.293
1.281
0.888
0.876
0.864
HO
10
9
7
11
12
N
N
O
O
O
HO
0.91
1.00
2.09
1.10
1.01
1.94
2.04
2.18
2.19
3.48
3.55
f1 (ppm)

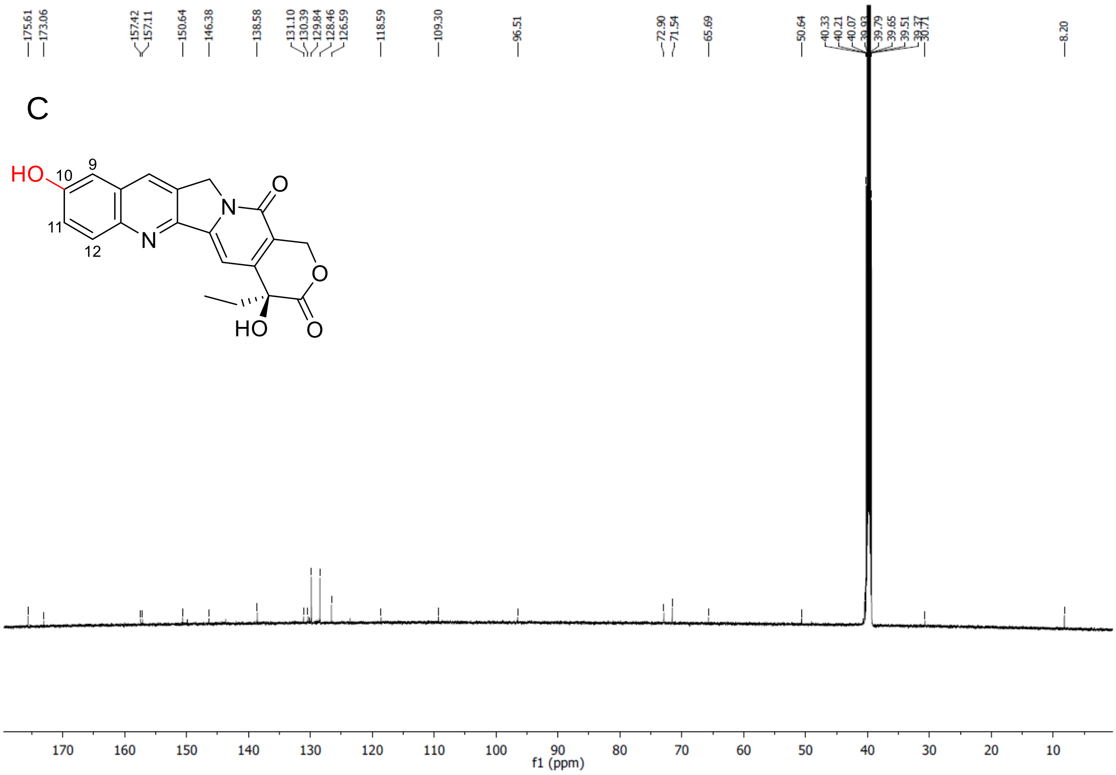

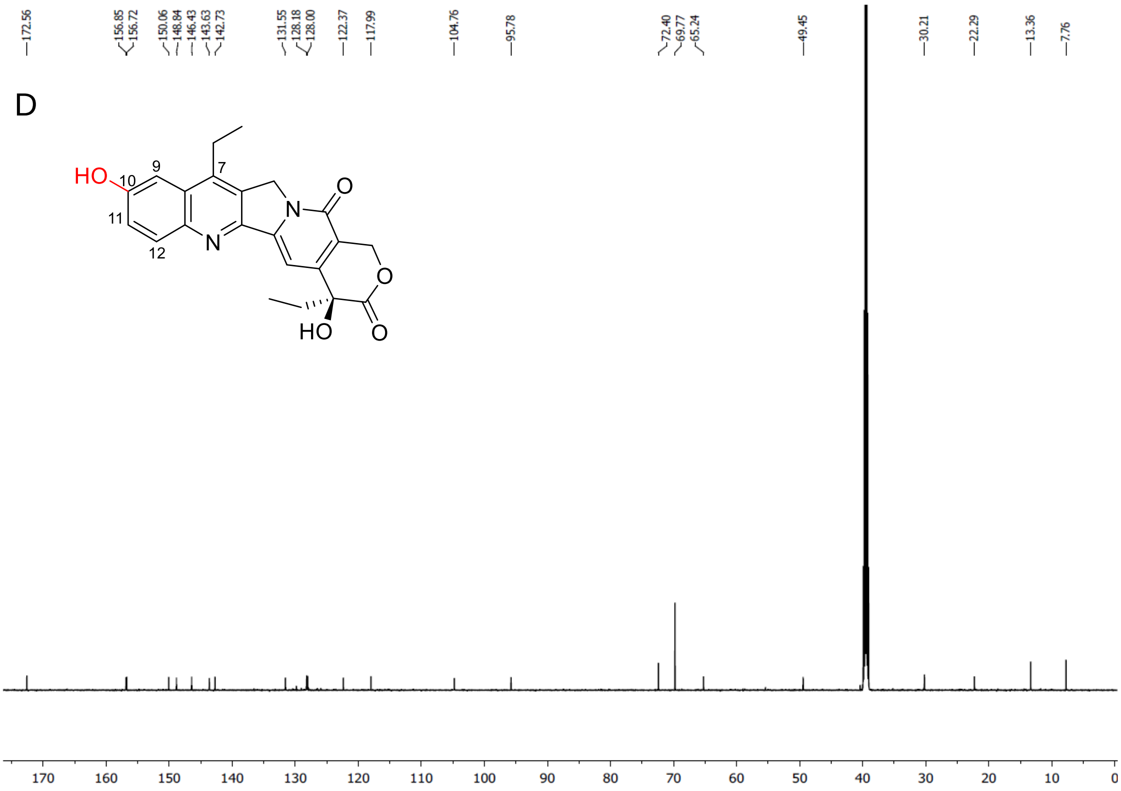

Supplementary Figure 4. ^{1}H NMR spectra products from *in vivo* assay of CaCYP32236/CPR with CPT producing 10HCPT **(A)**, and with 7-ethyl-CPT as substrate producing 7-ethyl-10HCPT (**B**). ^{13}C NMR spectra of 10HCPT **(C)** and 7-ethyl-10HCPT **(D)**.

A

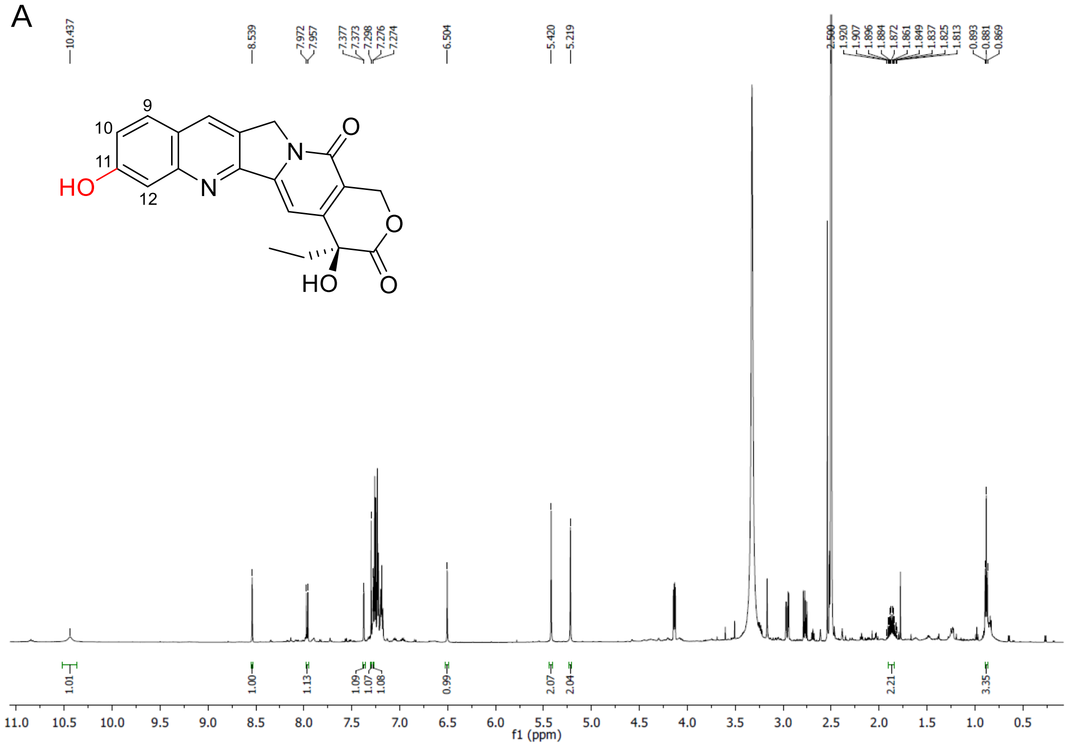

B

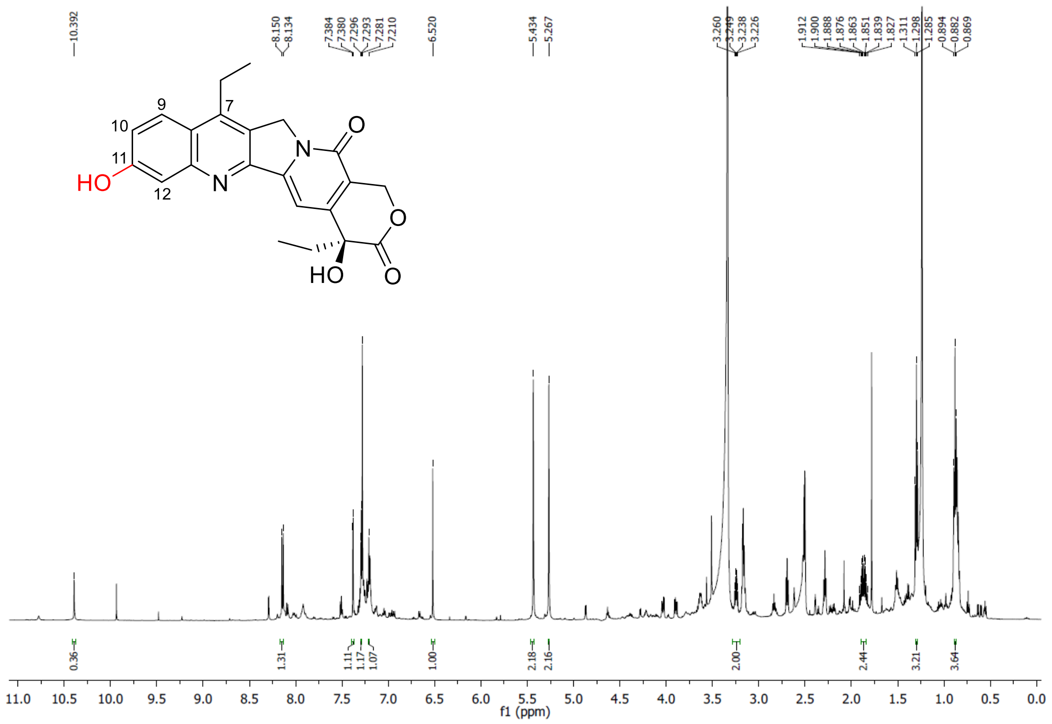

C

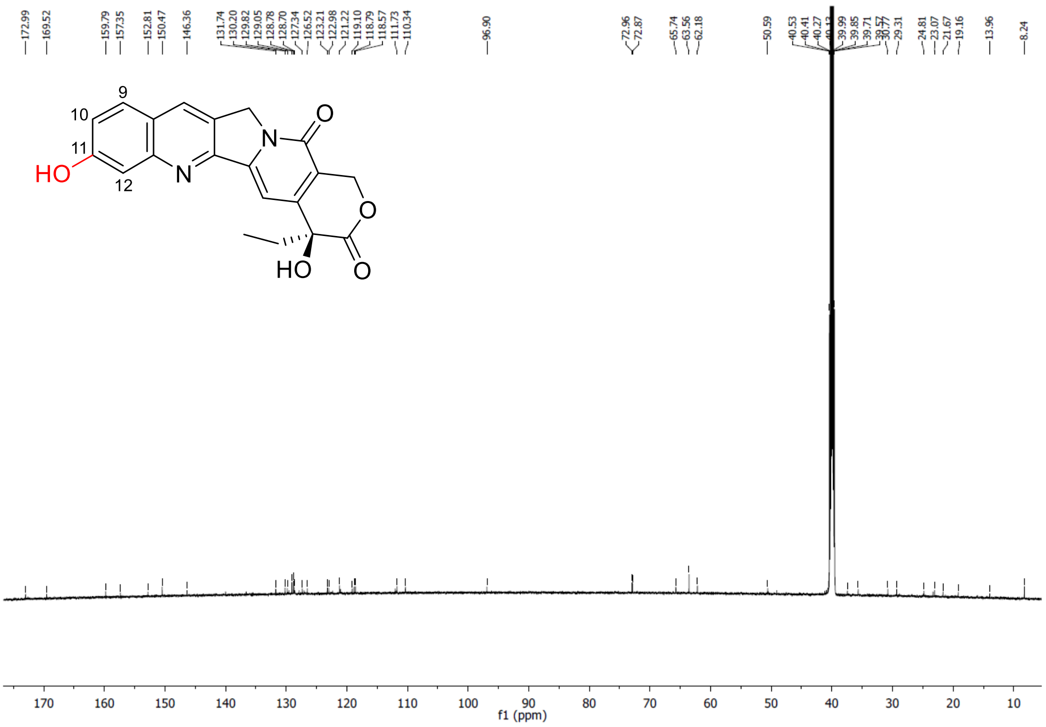

D

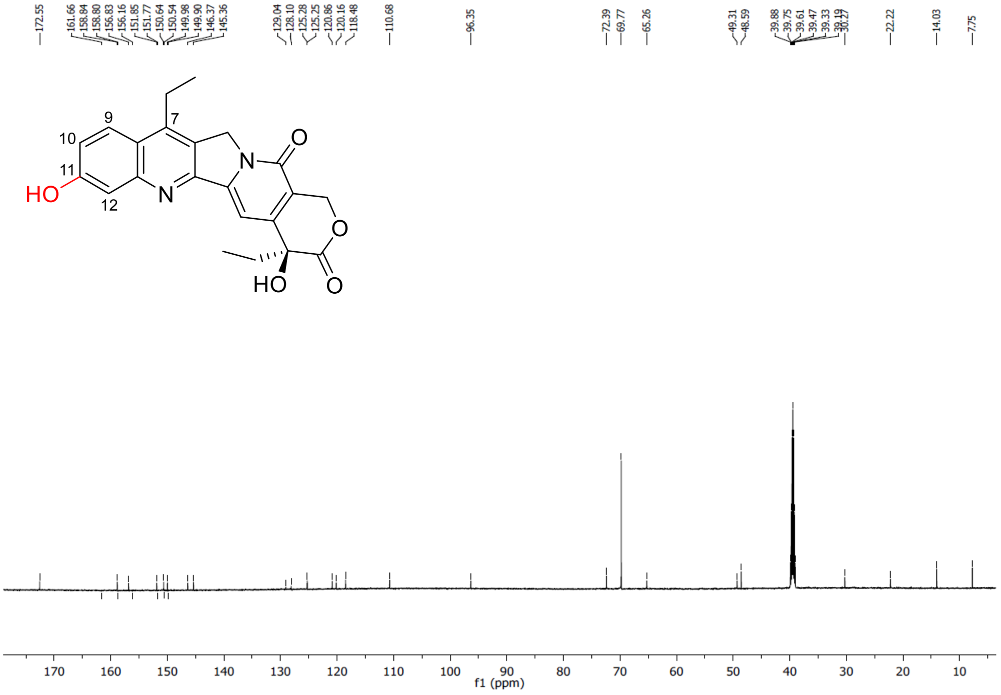

Supplementary Figure 5. ^{1}H NMR spectra of products from *in vivo* assay of Ca32229/CPR with CPT as substrate producing 11HCPT (**A**), and with 7-ethyl-CPT as substrate producing 7-ethyl-11HCPT (**B**). ^{13}C NMR spectra of 11HCPT **(C)** and 7-ethyl-11HCPT **(D)**.

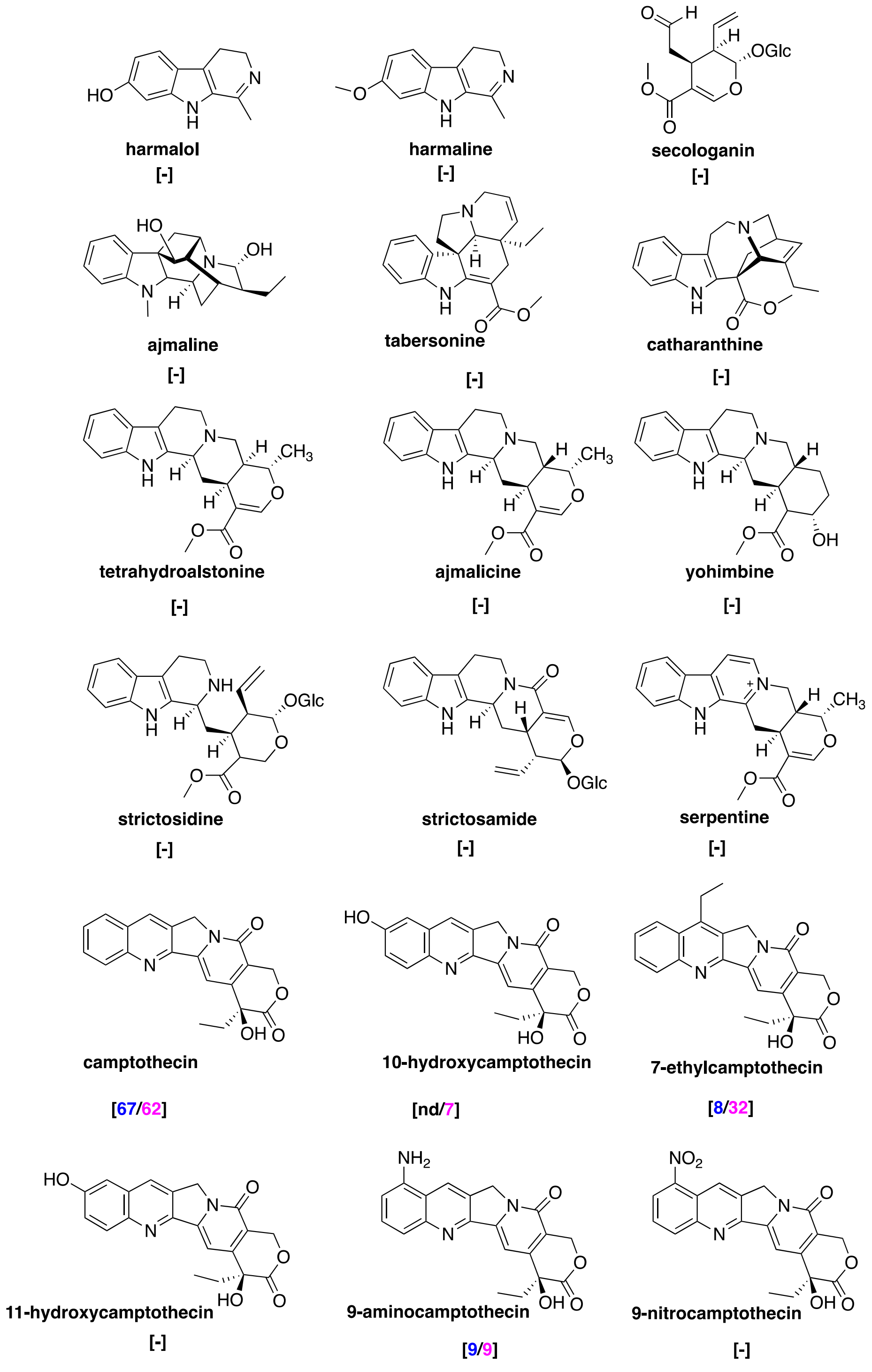

Supplementary Figure 6. Substrate specificity of CPTHs, Ca32236 (CPT 10-hydroxylase) and Ca32229 (CPT 11-hydroxylase). Substrates from different subgroups of monoterpenoid indole alkaloids (MIA) include simple secoiridoid (secologanin), central precursors of MIA biosynthetic pathway (strictosidine, strictosamide), heteroyohimbanes (ajmalicine, tetrahydroalstonine), yohimbane (yohimbine), ajmalan (ajmaline), β-carboline (harmalol, harmaline), and CPT and CPT analogues (10HCPT, 11HCPT, 7-ethyl-CPT, 9-amino-CPT, 9-nitro-CPT). Only CPT, 7-ethyl-CPT, 10HCPT, and 9-amino-CPT were accepted as substrates with different conversion rates. Numbers in brackets are conversion rates (blue for Ca32236 and pink for Ca32229), and [-] for non-detected rates.

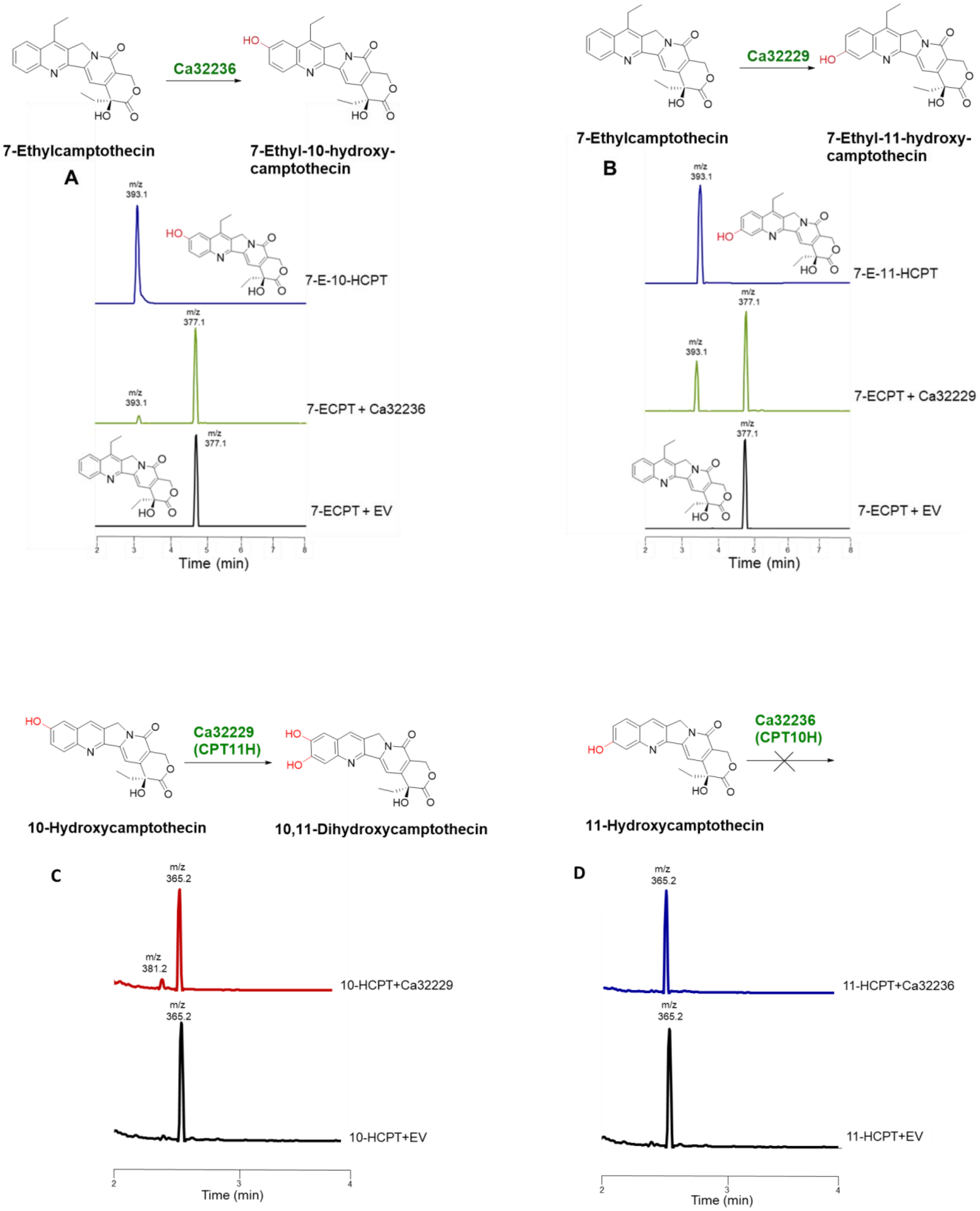

Supplementary Figure 7. Oxidation of 7-ethyl-CPT, 10HCPT and 11HCPT by Ca32229 and Ca32236. Extracted ion chromatograms showing the *in vivo* activity of Ca32236 (**A**) and Ca32229 (**B**) with 7-ethyl-CPT. CPT: camptothecin; HCPT: hydroxy-CPT; ECPT: ethyl-CPT; EV: empty vector (negative control). 10-HCPT can be further oxidized by Ca32229 (**C**) but not Ca32236 (**D**).

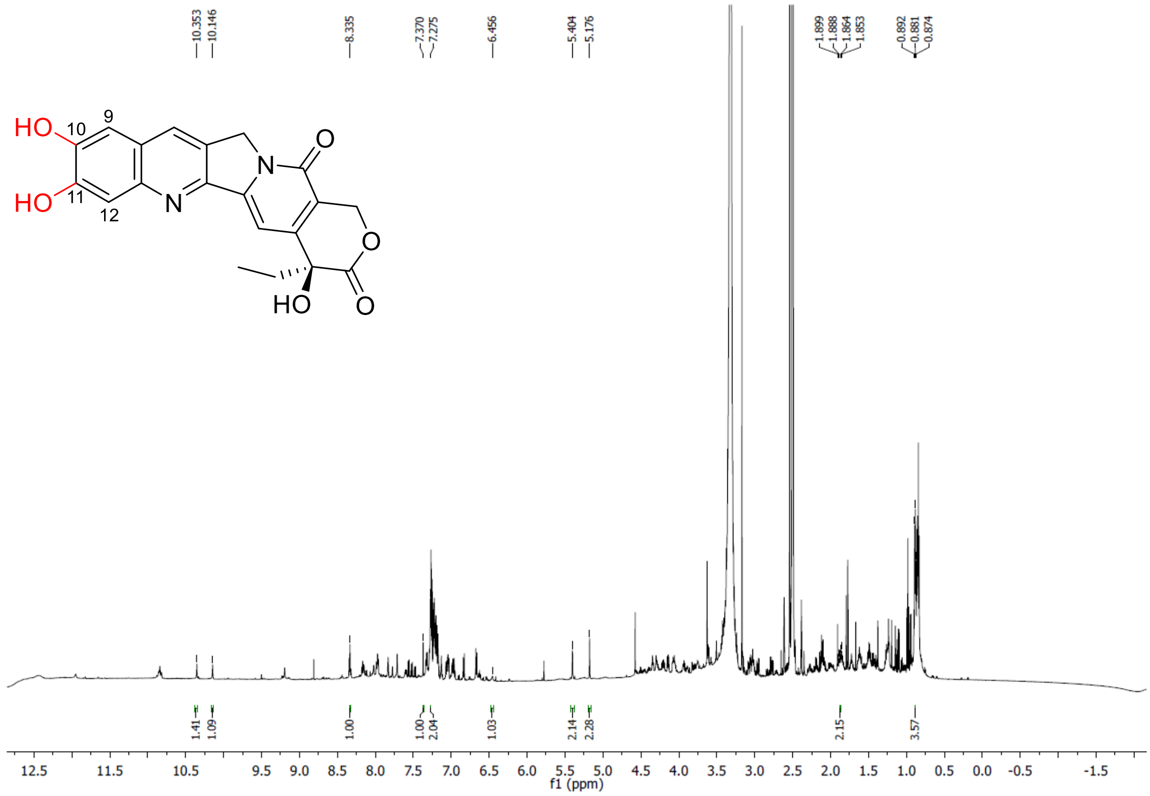

Supplementary Figure 8. ^{1}H NMR spectrum of products from *in vivo* assay of Ca32229/CPR with 10HCPT as substrate producing 10,11-dihydroxyCPT.

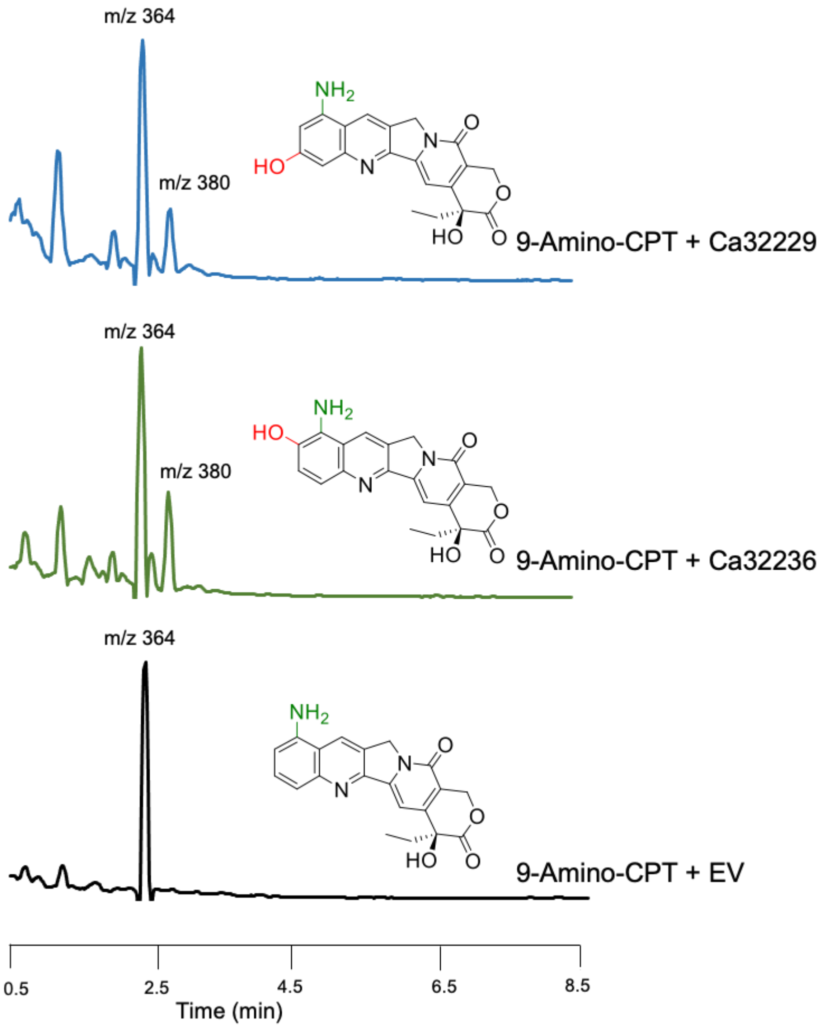

Supplementary Figure 9. Oxidation of 9-amino-CPT by Ca32229 produces 9-amino-11HCPT, and Ca32236 to produce 9-amino-10HCPT. Extracted ion chromatograms showing the *in vivo* activity of Ca32236 and Ca32229. 9-Amino-CPT: 9-aminocamptothecin; EV: empty vector (negative control). The hydroxylation positions were speculated based on the regio-specificity of Ca32229 and Ca32236 toward other substrates of the same scaffold.

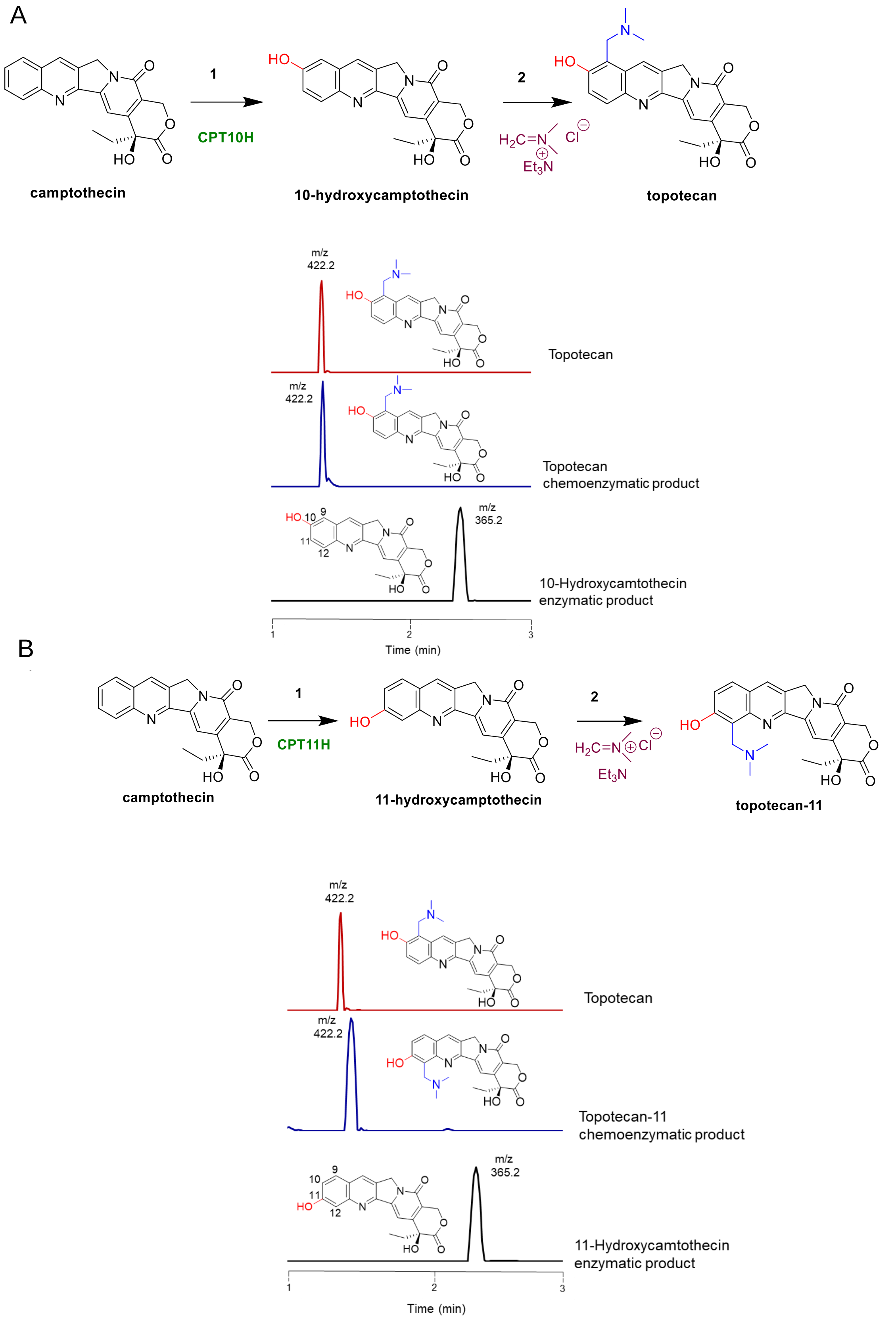

Supplementary Figure 10. Chemoenzymatic production of topotecan (**A**) and topotecan-11 (12-[(dimethylamino)methyl]-11HCPT) (**B**).

A

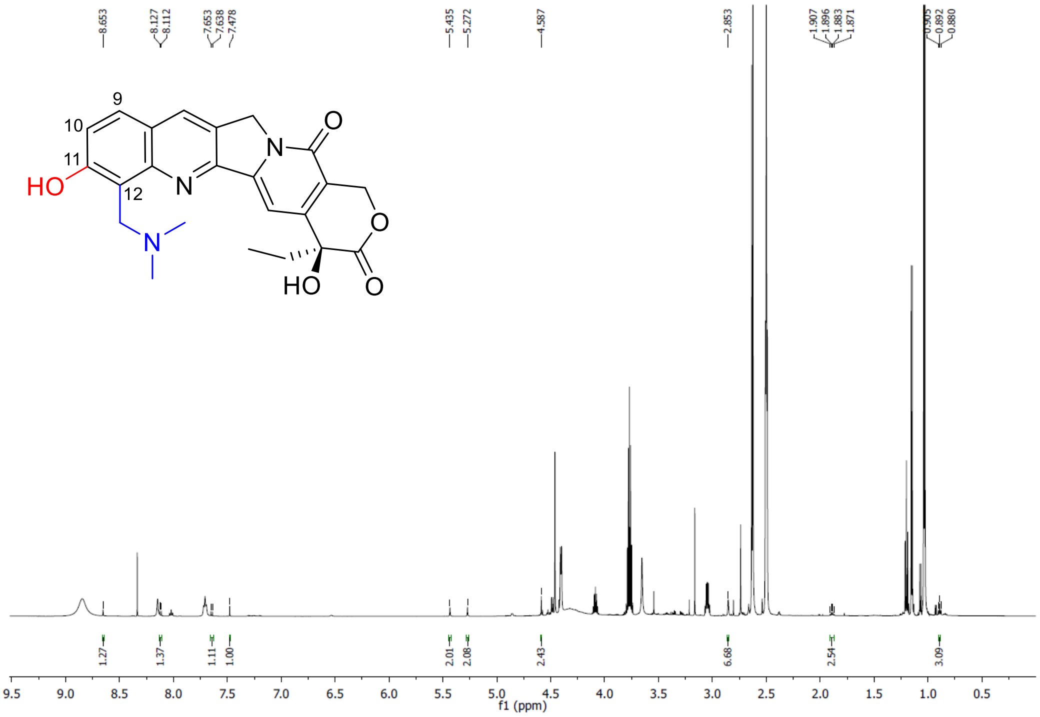

B

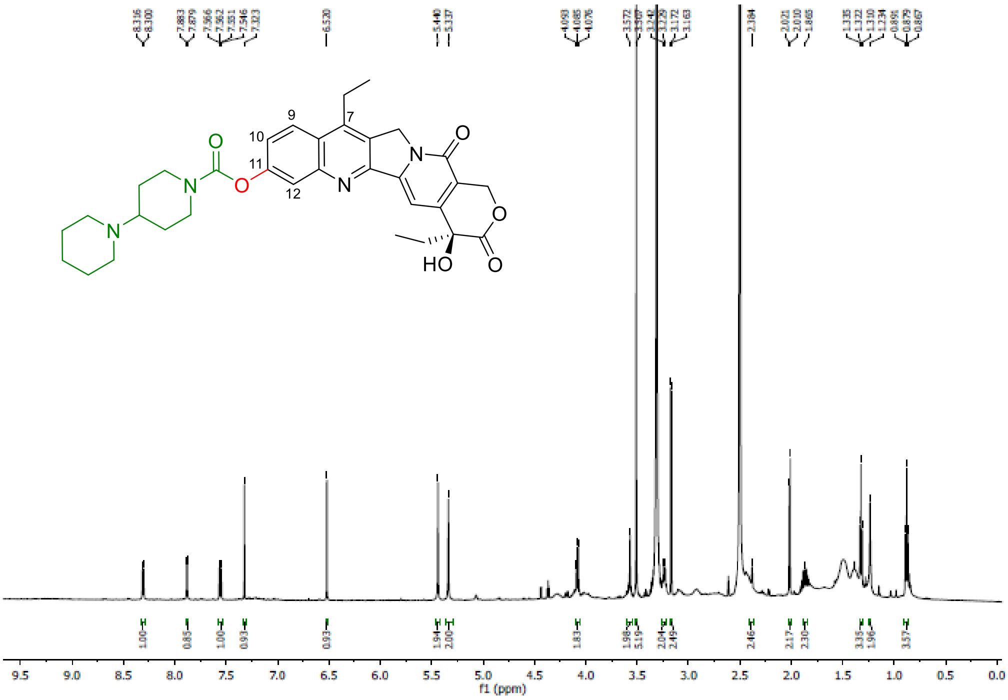

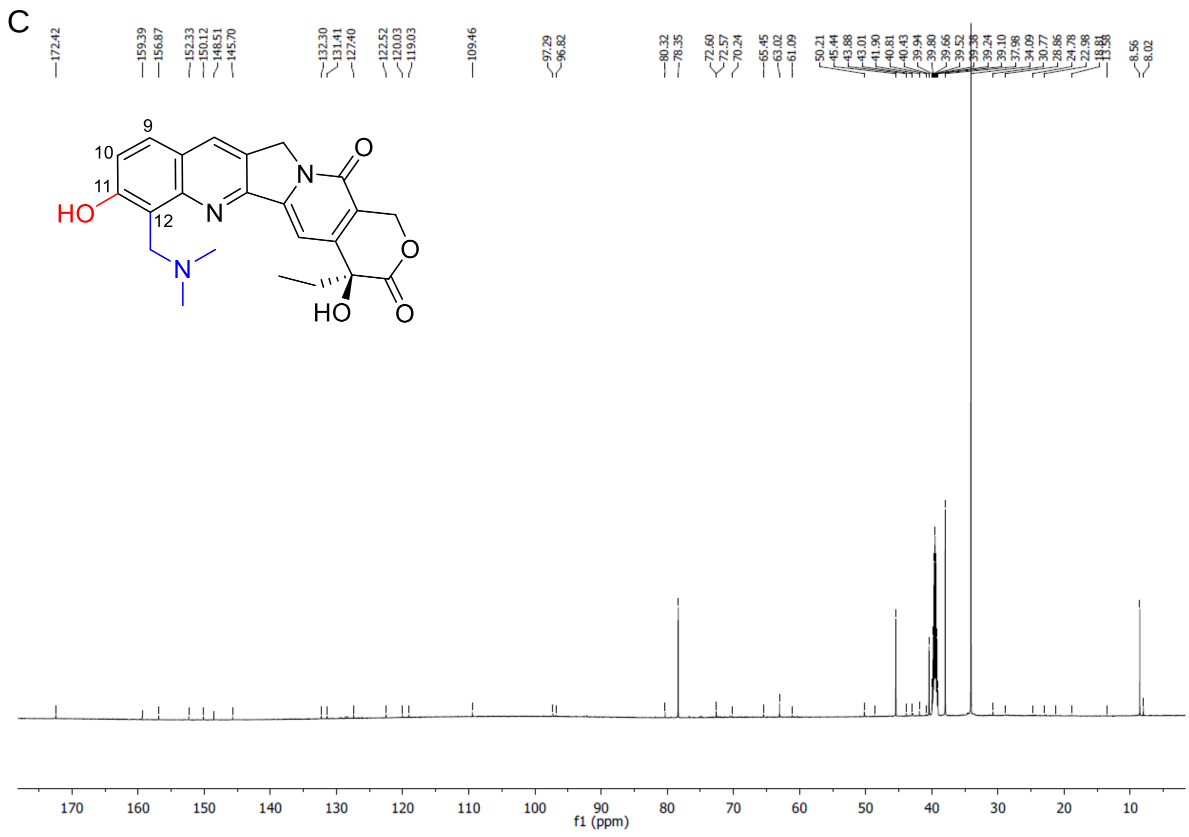

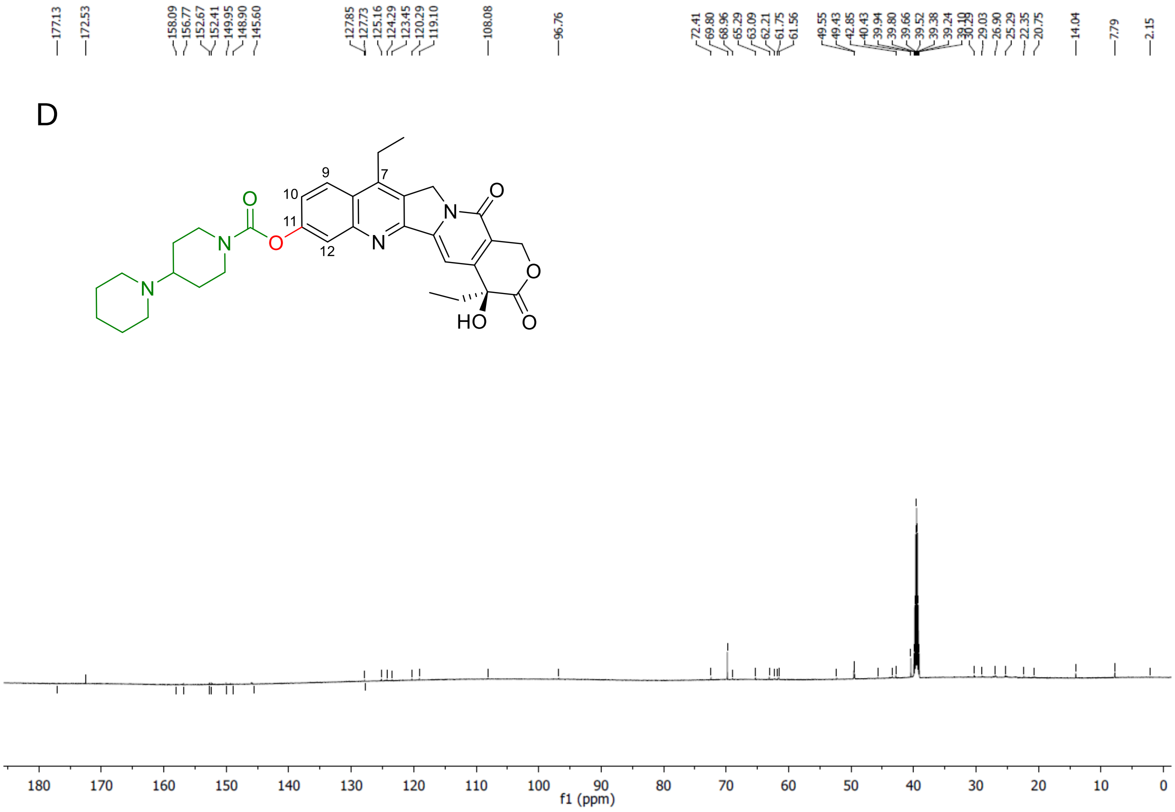

Supplementary Figure 11. ^{1}H NMR spectra of chemoenzymatic reaction products topotecan-11 (12-[(dimethylamino)methyl]-11HCPT) (**A**) and irinotecan-11 (7-ethyl-11-[4-(1-piperidino)-1-piperidino]carbonyloxyCPT) (**B**). ^{13}C NMR spectra of topotecan-11 **(C)** and irinotecan-11 **(D)**.

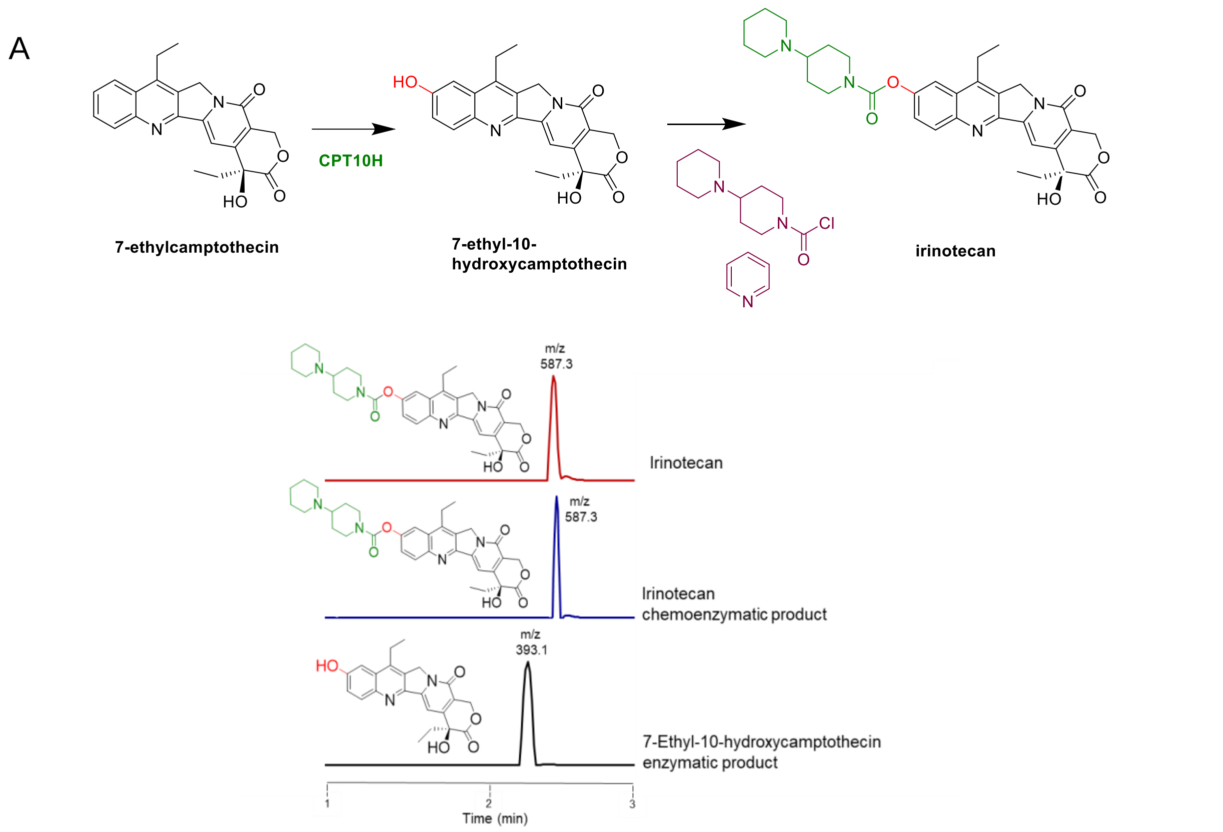

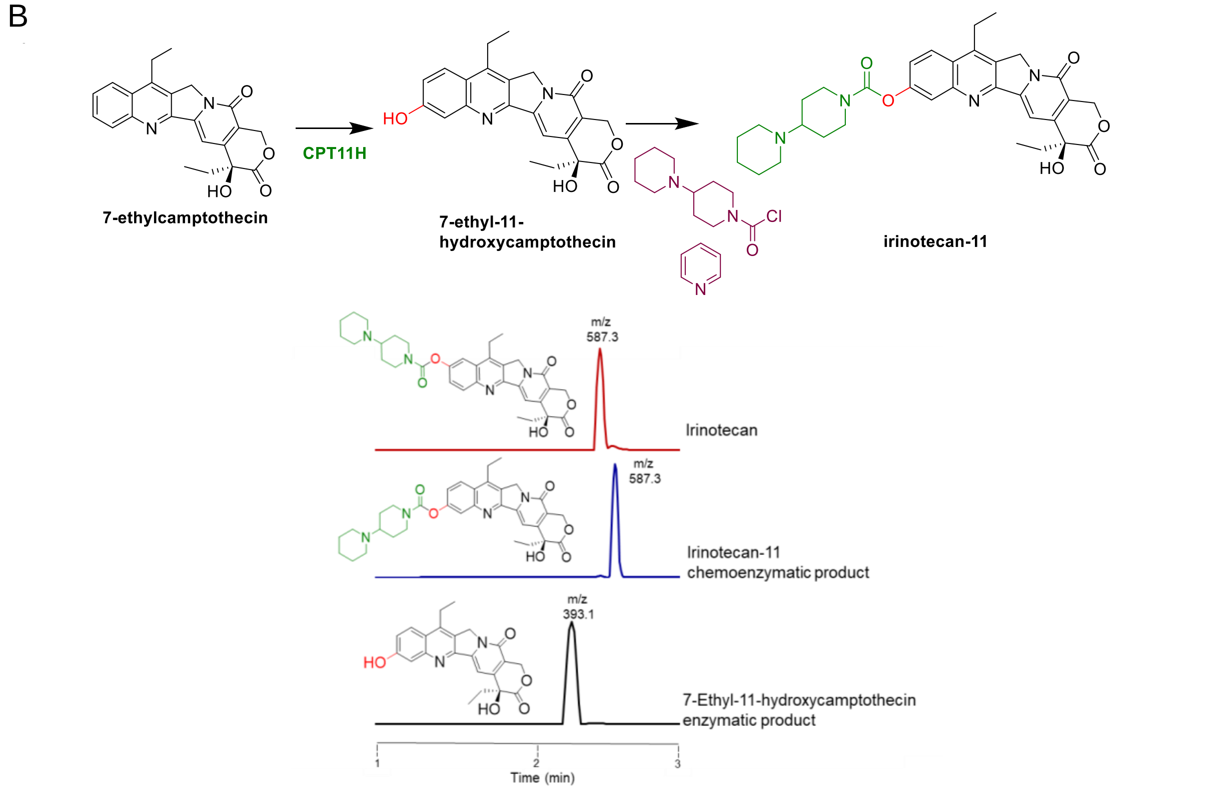

Supplementary Figure 12. Chemoenzymatic production of irinotecan (A) and irinotecan-11 (7-ethyl-11-[4-(1-piperidino)-1-piperidino]carbonyloxyCPT) (B).

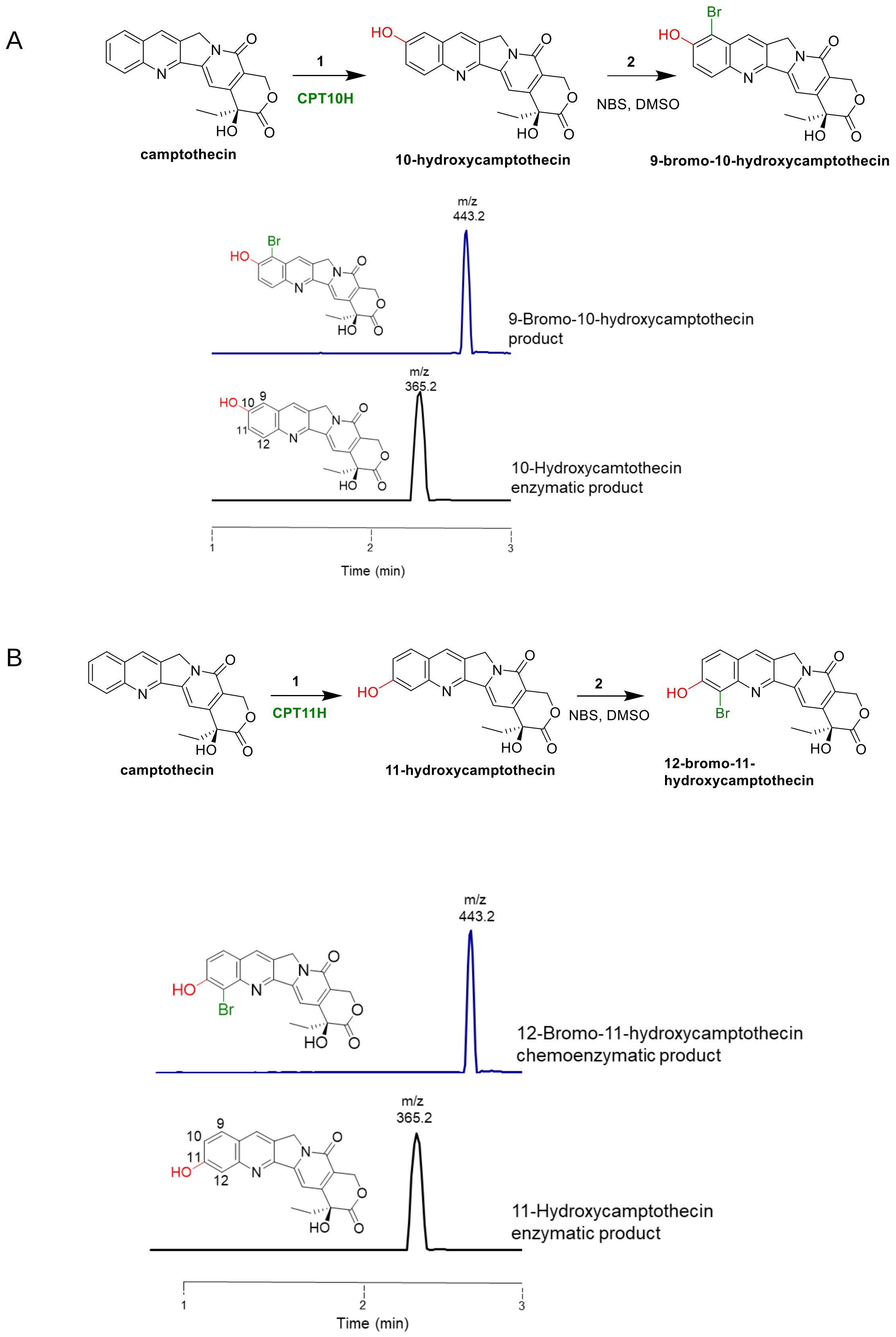

Supplementary Figure 13. Chemoenzymatic production of brominated HCPTs using CPT 10-hydroxylase (A) and CPT 11-hydroxylase (B) as biocatalysts.

A

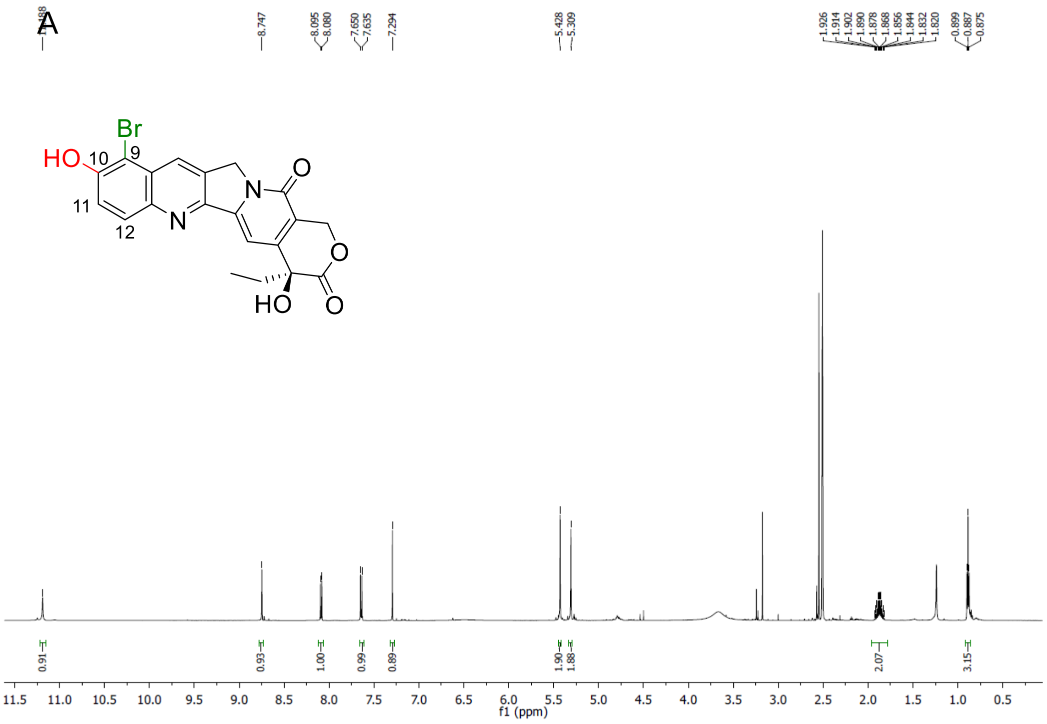

B

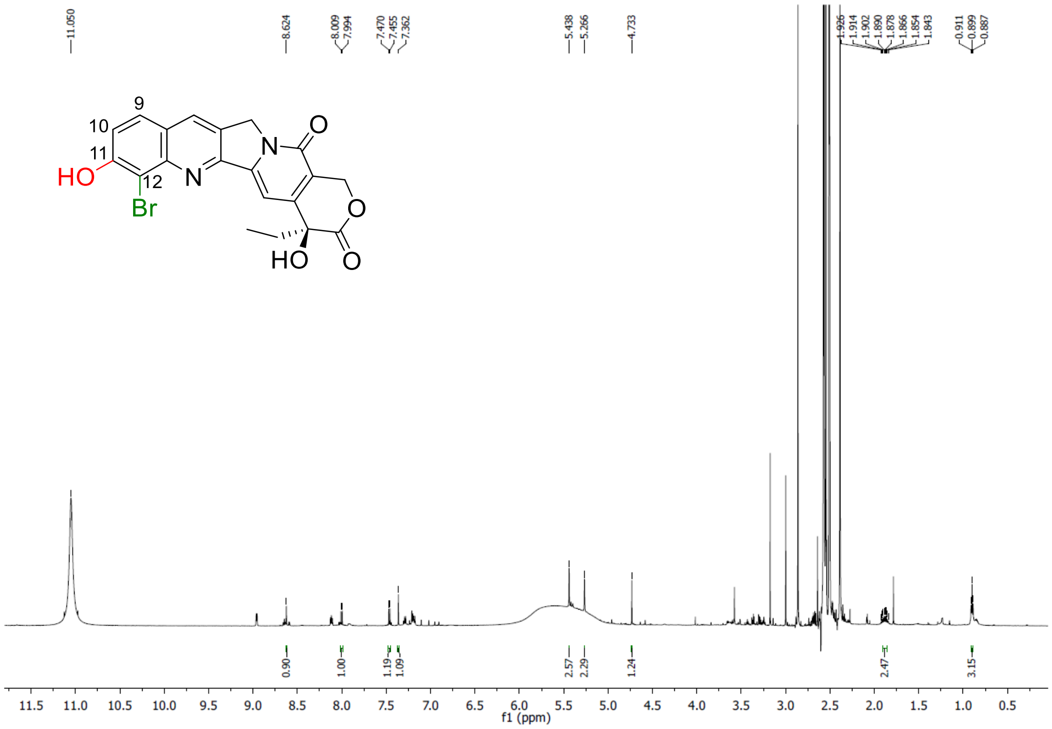

C

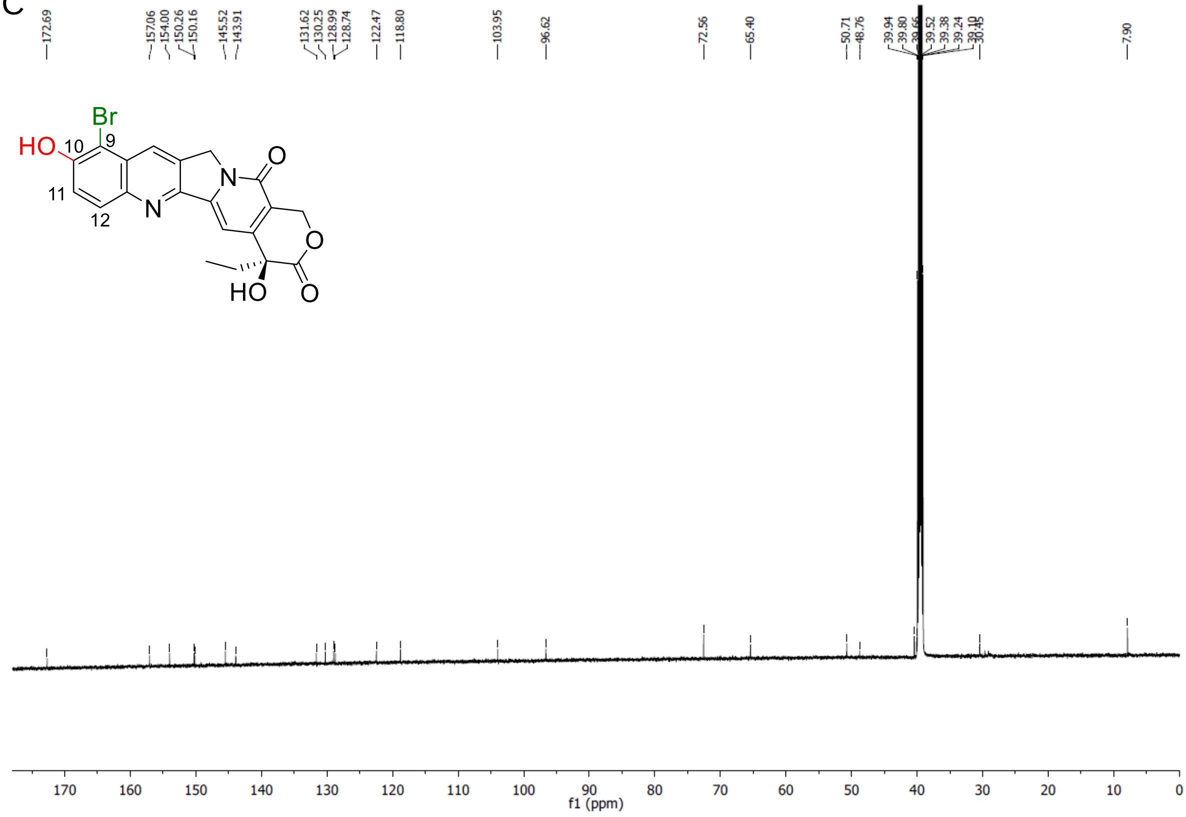

D

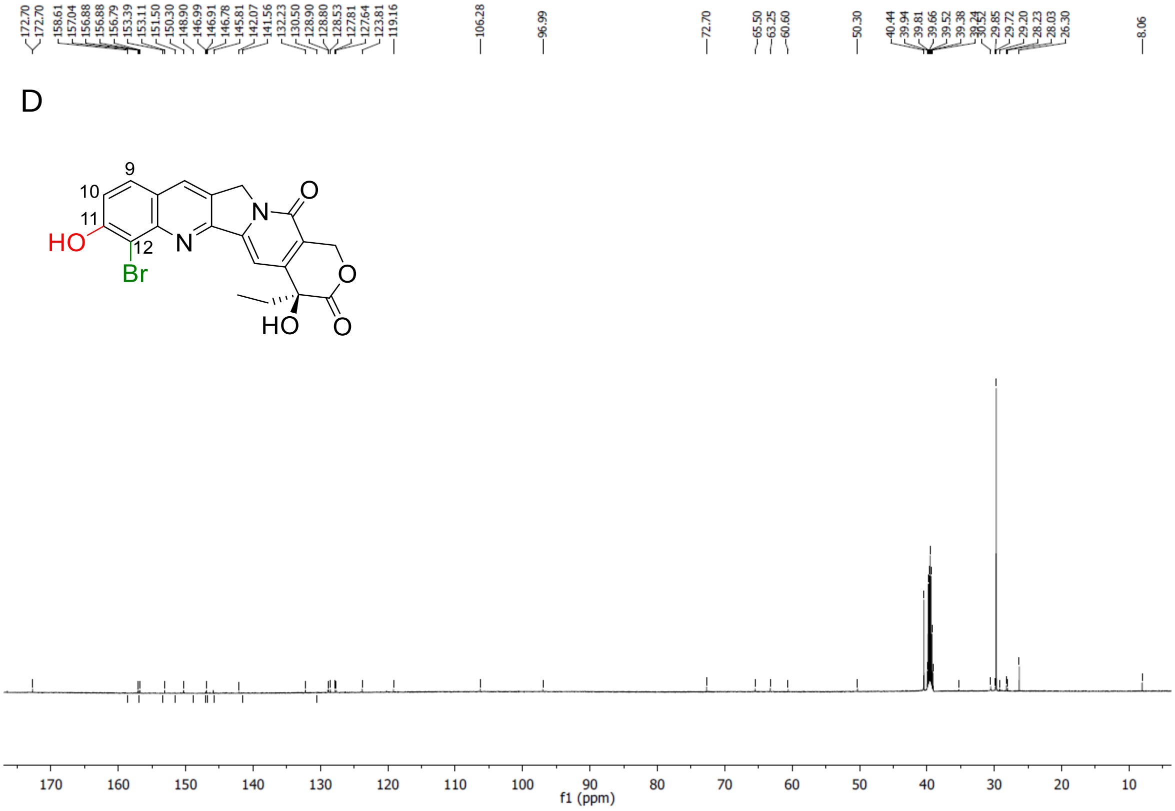

Supplementary Figure 14. ^{1}H NMR spectra of bromination reaction of 10HCPT as substrate producing 9-bromo-10HCPT (**A**), and of 11HCPT as substrate producing 12-bromo-11HCPT (**B**). ^{13}C NMR spectra of 9-bromo-10HCPT **(C)** and 12-bromo-11HCPT **(D)**.

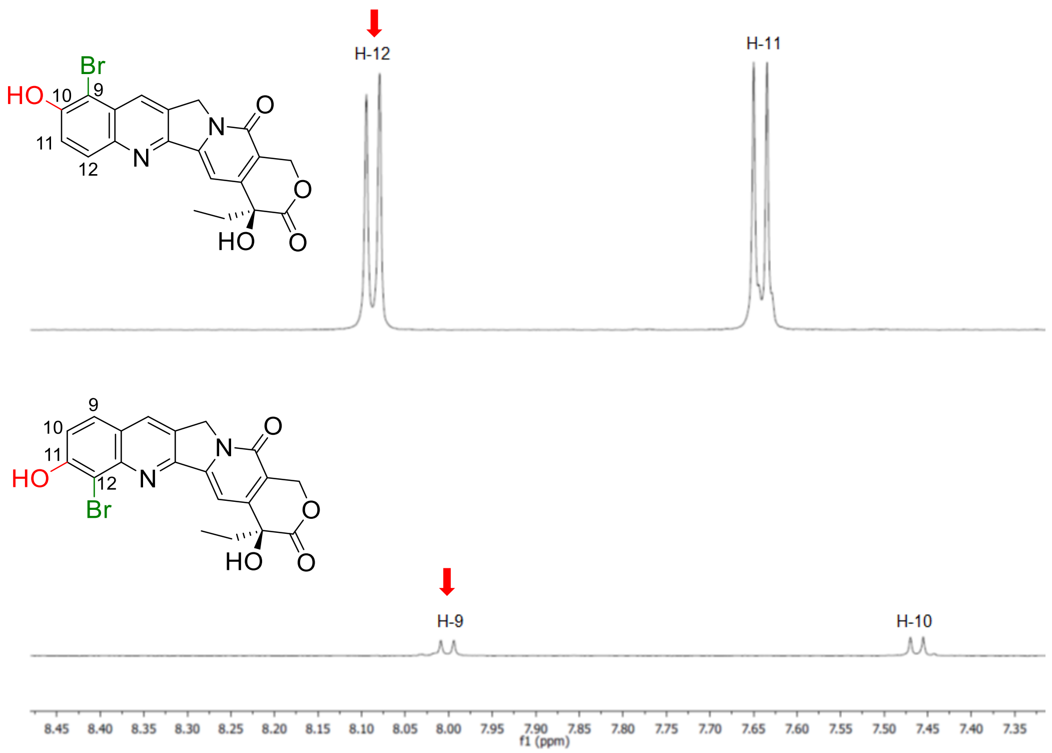

Supplementary Figure 15. 1D-TOCSY NMR spectra of brominated products of 10HCPT and 11HCPT

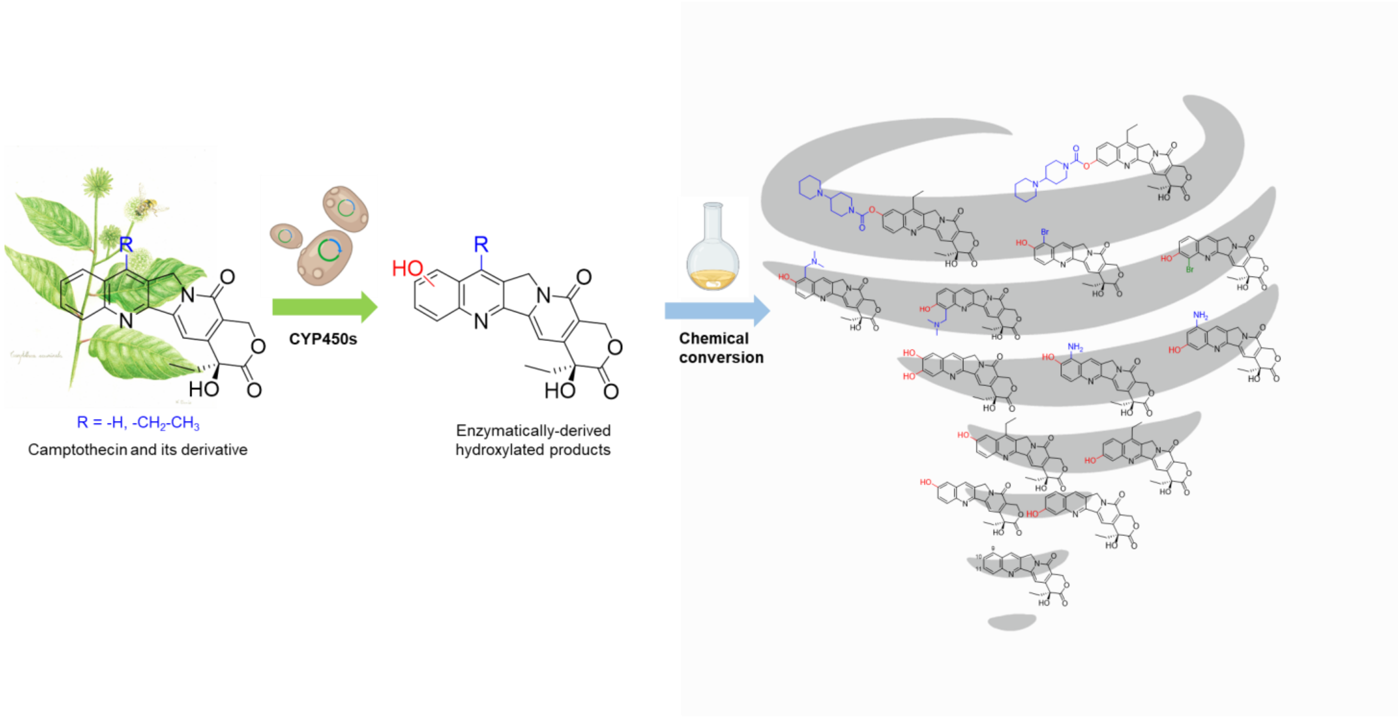

Supplementary Figure 16. CPT analogs produced by chemoenzymatic reactions of camptothecin hydroxylases (CPTHs). The *C. acuminata* artwork was created by K. Davis.

Supplementary Table 1. Summary of yeast *in vivo* assay yields of CPT hydroxylases with CPT-scaffold substrates

A. Product yield in whole-cell biotransformation of CPT and its analogues via CPTHs

Enzyme	Substrate	Product	Starting material	Conversion rate (%)[a]	Starting material	Yield of biotransformation in crude extract (mg)[b]	Pure products after semiprep HPLC (mg)
Ca32236/ CPT10H	CPT	10-HydroxyCPT	18.0	67	18.0	12.0	9.4
	7-EthylCPT	7-Ethyl-10CPT	18	8	18	1.5	0.6
	9-AminoCPT	9-Amino-10CPT	18	9	18	1.7	n/a[c]
Ca32229/ CPT11H	CPT	11-HydroxyCPT	17	62	17	11.0	8.1
	7-EthylCPT	7-Ethyl-11-hydroxyCPT	19	32	19	6.1	3.5
	9-AminoCPT	9-amino-10-hydroxyCPT	18	9	18	1.7	n/a[c]
	10-HydroxyCPT	10,11-DihydroxyCPT	18	11	18	2.0	0.6

[a]conversion rate calculated based on LCMS analysis
[b]yield of biotransformation from the yeast *in vivo* assay was obtained from 1 L yeast culture incubated with 17 mg CPT starting material.
[C] due to the low yield and low recovery rate of our semi prep system, these products couldn't be recovered for further structural elucidation

B. Product yield in semisynthesis of new compounds from enzymatic products

Enzymatic products	Semisynthetic products	Starting material (mg)	Conversion rate (%)[a]	Yield of semi-synthetic products (mg)	Product recovery (mg) after semiprep HPLC
10-Hydroxycamptothecin	Topotecan	6.9	100	8	4.0
	9-bromo-10HCPT	10.5	100	12.75	4.0
7-Ethyl-10-hydroxycamptothecin	Irinotecan	1.1	100	1.7	1.5
11-Hydroxycamptothecin	Topotecan-11	5.9	100	6.8	6.0
	12-bromo-11HCPT	3.1	100	3.8	1.1
7-Ethyl-11-hydroxycamptothecin	Irinotecan-11	7.4	100	11.5	8.0

Supplementary Table 2. Primers used for the construction of expression vectors

Primers used to assemble CYP450 candidates in pESC-leu2d expression vector

Vector name	Forward primer (5' to 3')	Reverse primer (5' to 3')	Insert size (bp)
pESC-Leu2d-32245	CAC TAA AGG GCG GCC AAC AAA ATG GAG AAG TTG TAC TAC TGC	CAC TAA AGG GCG GCC AAC AAA ATG GAG AAG TTG TAC TAC TGC CT	1542
pESC-Leu2d-12175	CAC TAA AGG GCG GCC AAC AAA ATG GAG TGG ACA TTG AGC ACA C	ATC CAT CGA TAC TAG TGC AAA CAC TCC TCG TCT	1554
pESC-Leu2d-17987	CAC TAA AGG GCG GCC AAC AAA ATG GAT TTC TTT AGC TTT TTG TTG	ATC CAT CGA TAC TAG AAC AAG GGT TGG AAC AGC ACG	1494
pESC-Leu2d-32709	CAC TAA AGG GCG GCC AAC AAA ATG GAG TGG ACA TTG AGC ACA	ATC CAT CGA TAC TAG TGC AAA CAC TCG TCG TCT TTT G	1554
pESC-Leu2d-32236	CAC TAA AGG GCG GCC AAC AAA ATGGAGAACTTGTACTACTGCCT	ATC CAT CGA TAC TAG ACGGAAACAAGTGCCTTCA	1533
pESC-Leu2d-32245	CAC TAA AGG GCG GCC AAC AAA ATG GAG AAG TTG TAC TAC TGC	ATC CAT CGA TAC TAG AATAAAGGAAGTGTCTTCAAGCTGG	1542
pESC-Leu2d-15664	CAC TAA AGG GCG GCC AAC AAA ATG ACT TGG TTT CTC TTT CTT CTT GC	ATC CAT CGA TAC TAG GAG TTT GTG TAA AAT CAA GTG AGC AC	1509
pESC-Leu2d-32229	CAC TAA AGG GCG GCC AAC AAA ATGGAGAACTTGTACTACTGCCT	ATC CAT CGA TAC TAG ACTGAAACAAGTGTCTTCAAGCTG	1536
pESC-Leu2d-15529	CAC TAA AGG GCG GCC AAC AAAATGGCTCCCCTTCTACTGCT	ATC CAT CGA TAC TAG TTC AAC GAT TGA AGG AAC AT	1518
pESC-Leu2d-12663	CAC TAA AGG GCG GCC AAC AAA ATG GAG ATG GAA ATA ATG AAC TCA	ATC CAT CGA TAC TAG GAGTTTGTGTAAAATCAAGTGAGCAC	1563

SUPPLEMENTARY REFERENCES

1. Hu, T.-C. & Harn, P.-J. PROCESS FOR MAKING TOPOTECAN. vol. 2 (2011).
2. Luo, Y., Yu, S., Huang, Q. & Lu, W. Synthesis of 9-Allyl-10-hydroxycamptothecin via Suzuki Reaction. *Journal of Heterocyclic Chemistry* **51**, 1133–1136 (2014).
